# Prognostic Value of the Charlson Comorbidity Index for Mortality and Machine Learning–Based Prediction in Critically Ill Patients with Paralytic Ileus: Retrospective Cohort Study

**DOI:** 10.2196/76003

**Published:** 2025-10-16

**Authors:** Hui Feng, Fuhai Zhou, Yi Shen, Zhen Wang, Yiyang Yuan, Wenshan Jing, Zhou Zheng, Hui Peng, Qingsheng Yu

**Affiliations:** 1 Department of Emergency Surgery, The First Affiliated Hospital of Anhui University of Chinese Medicine, Hefei, China; 2 Institute of Surgery, Anhui Provincial Academy of Traditional Chinese Medicine, Hefei, China; 3 Department of General Surgery, The First Affiliated Hospital of Anhui University of Chinese Medicine, No. 117 Meishan Road, Hefei, 230038, China, 86 055162850183

**Keywords:** Charlson Comorbidity Index, paralytic ileus, MIMIC-IV database, all-cause mortality, machine learning

## Abstract

**Background:**

The burden of paralytic ileus (PI) in the intensive care unit remains high, and the Charlson Comorbidity Index (CCI) is strongly associated with the prognosis of several acute and chronic diseases. However, evidence specifically evaluating the prognostic value of CCI in intensive care unit patients with PI remains limited.

**Objective:**

This study aimed to investigate the association between CCI and clinical prognosis in critically ill patients with PI.

**Methods:**

In this study, data were extracted from the Medical Information Mart for Intensive Care IV (version 2.2), a large, publicly available critical care database, and used to determine the optimal cut-off value of CCI for predicting mortality in patients with PI using the receiver operating characteristic curves, and the association between CCI and mortality was evaluated using Cox regression and restricted cubic spline analysis. A machine learning (ML) prediction model was then constructed to predict hospital mortality by combining CCI and other clinical characteristics.

**Results:**

The study included 863 patients with PI (age: median 65.4, IQR 54.6‐75.5 y; male: 575/863, 66.6%). The receiver operating characteristic curve identified an optimal cut-off value of 4.5 for CCI. The multivariate Cox regression analysis showed that compared to the lowest CCI quartile, patients with elevated CCI levels were more likely to have elevated hospital (Q4: hazard ratio [HR] 2.447, 95% CI 1.210‐4.951), 28-day (Q4: HR 3.891, 95% CI 1.956‐7.740), and 90-day (Q4: HR 3.994, 95% CI 2.224‐7.173) all-cause mortality were significantly associated with elevated CCI levels; however, the association with ICU mortality (Q4: HR 1.892, 95% CI 0.653‐5.480) was weak. Among the 11 ML models, the light gradient boosting machine model performed best, with internal validation results showing an area under the curve of 0.811, a geometric mean of 0.670, and an *F*_1_-score of 0.895.

**Conclusions:**

The CCI is an important predictor of hospital, 28-day, and 90-day all-cause mortality in critically ill patients with PI, and the optimal threshold is 4.5. ML models, including the CCI, show high accuracy in predicting hospital mortality, and the CCI occupies an important position in the model. This suggests that the CCI helps to identify high-risk patients, supports clinical decision-making, and improves prognosis.

## Introduction

### Background

Paralytic ileus (PI) is a common functional intestinal obstruction due to decreased or loss of intestinal motility, with etiological factors including electrolyte imbalance, severe infection, substance abuse, and abdominal surgery [1,2]. The global incidence is approximately 1‐5 cases per 100,000 people, with a higher incidence in women but a higher mortality rate in men [3]. The overall mortality rate for PI is 5%‐6%, but this increases to 10%‐20% in the intensive care unit (ICU) patients [1,4]. Patients with PI are often associated with chronic diseases or comorbidities such as diabetes, neurological diseases, systemic sclerosis, and malignant cancer, and the cumulative burden of these underlying diseases may have a significant impact on mortality [5-8]. Given the high morbidity and mortality associated with PI, it is important to accurately identify high-risk patients and assess their prognostic risk.

The Charlson Comorbidity Index (CCI) is a widely used scoring system that quantifies the burden of chronic comorbidities (eg, diabetes, malignant cancer, and connective tissue disorders) and evaluates their impact on overall prognosis, thereby providing an estimate of mortality risk [9,10]. It has demonstrated significant prognostic value across a variety of clinical scenarios, including older patients, critically ill populations, and cancer cohorts [11-14], and has been validated in settings such as chronic granulocytic leukemia [15], the assessment of postoperative complications [16], and fracture risk prediction [17]. These findings highlight the use of the CCI in capturing the cumulative effect of chronic disease burden on patient outcomes. In critically ill patients with PI, comorbidities such as cardiovascular disease, diabetes, and chronic kidney disease are common and may substantially influence prognosis, making the CCI particularly relevant for this cohort [1,4]. Moreover, unlike scoring systems such as Sequential Organ Failure Assessment (SOFA) or Acute Physiology and Chronic Health Evaluation that primarily reflect acute illness severity, the CCI captures the cumulative impact of chronic conditions on outcomes, thereby complementing conventional acute severity scores [9]. However, evidence specifically evaluating the prognostic value of the CCI in ICU patients with PI remains limited.

### Objective

With the rapid development of big data and machine learning (ML), mortality prediction models based on electronic health records have become a research focus, as ML can capture complex associations in high-dimensional data and often outperform traditional methods [18-20]. Recently, explainable ML approaches have been applied in critical care, with several studies using the Medical Information Mart for Intensive Care (MIMIC) database to develop interpretable mortality prediction models that enhance clinical applicability [21,22]. For instance, a deep-learning model for mortality prediction based on MIMIC used neural networks with Shapley Additive Explanations (SHAP) analysis to predict outcomes in ICU patients with PI [23]. Building on these advances, the primary objective of this study was to evaluate the association between CCI and all-cause mortality, including hospital, ICU, 28-day, and 90-day mortality, in ICU patients with PI. The exploratory objective was to assess the feasibility of incorporating CCI into ML models for mortality prediction in this population.

## Methods

### Study Participants

This is a retrospective cohort study. The Massachusetts Institute of Technology and Beth Israel Deaconess Medical Center (BIDMC) collaborated to develop the MIMIC-IV electronic database (version 2.2), which was used for this study [24]. The database contains information on more than 50,000 patients who received ICU care at BIDMC between 2008 and 2019. The database provides a wide range of clinical data and has been used extensively in critical care medicine research [25-27]. Detailed information about the database, including access instructions, is available at PhysioNet [28]. The institutional review board (IRB) of BIDMC waived informed consent and allowed sharing of research resources because all data were deidentified. Therefore, this study did not require additional informed consent (2001-P-001699/14; 0403000206). To comply with the regulations, the author, HF, obtained a Cooperative Institutional Training Initiative license (certification: 61863057) and the necessary permission to use the MIMIC-IV database.

Adult patients who were first admitted to the hospital with a diagnosis of PI according to the International Classification of Diseases (ICD-9: 560.1 and ICD-10: 56.0) were included in this study. Individual patient consent was not required due to the anonymized nature of patient health information in this database. Exclusion criteria included: (1) patients who stayed in the ICU for <6 hours; (2) multiple ICU admissions for which only data from the first admission were extracted; and (3) patients who lacked information on CCI. Finally, a total of 863 patients were included in this study and divided into 4 groups according to the quartiles of CCI.

### Patient Characteristics

Data collection involved the use of structured query language and PostgreSQL (version 16.3.7; PostgreSQL Global Development Group) to extract baseline patient characteristics. The following data were obtained: (1) demographic information includes sex, age, and race; (2) comorbidities identified according to ICD-9 or ICD-10: congestive heart failure, renal disease, malignant cancer, sepsis, diabetes, and hypertension; (3) mean vital signs include respiratory rate (RR), mean blood pressure (MBP), heart rate, and oxygen; (4) laboratory parameters include white blood cell (WBC) count, red blood cell distribution width (RDW), platelets, hemoglobin, anion gap, blood urea nitrogen (BUN), creatinine, total calcium, chloride, sodium, and potassium; (5) severity scores include Simplified Acute Physiology Score II (SAPS II), Oxford Acute Severity of Illness Score (OASIS), Acute Physiology Score III (APS III), SOFA score, and CCI; and (6) therapeutic agents include ondansetron and neostigmine. All laboratory variables were obtained from the first 24 hours after the patient’s admission to the ICU. Follow-up began on the date of ICU admission and ended on the date of death. Variables with more than 10% missing values were excluded to mitigate potential bias [29]. The proportions of missing values for variables are shown in Figure S1A in Multimedia Appendix 1, and variables with less than 10% missing values were interpolated using the random forest interpolation method implemented in the *missForest* package of the R software (R Foundation for Statistical Computing) [30]. In the ML section, the patients with PI were divided into a 70% training cohort and a 30% test cohort.

### Clinical Outcome

The primary outcome of this study was all-cause hospital mortality, and secondary outcomes were ICU mortality and mortality at 28 and 90 days after ICU admission. In addition, the primary objective of the ML modeling was all-cause hospital mortality.

### Statistical Analysis

The normality of continuous variables was assessed using the Kolmogorov-Smirnov test. Continuous variables that followed a normal distribution were analyzed using the 2-tailed *t* test or ANOVA, and those that were not normally distributed were analyzed using the Mann-Whitney *U* test or the Kruskal-Wallis test. Categorical variables were compared by the chi-square test. The CCI was categorized into 4 quartiles, Kaplan-Meier survival analysis was used to assess differences in mortality between groups, and log-rank tests were used to compare differences between groups. The optimal cut-off value for CCI was determined by receiver operating characteristic (ROC) curve analysis using the Youden index maximization method. Variance inflation factor (VIF) was used to assess multicollinearity between variables, and variables with a VIF>5 were removed (Figure S1B in Multimedia Appendix 1).

Cox regression analysis was used to assess the association between CCI and hospital mortality, ICU mortality, as well as 28- and 90-day mortality, with model 1 including only CCI and model 2 including age and sex as correction variables. Model 3 was developed based on clinical expertise, a previous study by Zhao et al [4], and the results of feature importance selection from the least absolute shrinkage and selection operator (LASSO) algorithm. The following variables were included: sex, age, CHF, renal disease, malignant cancer, sepsis, diabetes, hypertension, RR, SpO₂, WBC, RDW, platelets, anion gap, BUN, creatinine, SAPS II, OASIS, APS III, and SOFA. In addition, restricted cubic spline (RCS) analyses were performed to explore the potential nonlinear relationship between CCI levels and hospital, ICU, and 28- and 90-day all-cause mortality rates. The CCI was used as a continuous or ordinal variable entered into the model (the first quartile of CCI served as the reference group). *P* values for trends were calculated using IQRs. Further stratified analyses were performed according to sex, age (≤65 and >65 y), CHF, malignant cancer, sepsis, and diabetes to determine the consistency of the prognostic value of CCI for the primary outcome. Likelihood ratio tests were used to examine the interaction between CCI and the variables used for stratification. A 2-sided *P*<.05 was considered statistically significant.

To test the robustness of the results of the primary analysis, several sensitivity analyses were performed. First, patients who died within 3 days of ICU admission were excluded to assess whether these patients had a significant impact on the primary outcome. Second, patients were grouped according to the optimal cut-off value of the CCI to test the stability of the main results by different CCI groupings. Finally, a stratified analysis of SOFA scores was performed: patients were divided into 3 groups based on their 24-hour SOFA scores after ICU admission: low (first 1/3 quartile), intermediate (1st or 3rd to 2nd or 3rd quartile), and high (last 1/3 quartile). This grouping method helps to further validate the effect of different SOFA score groups on the association between CCI and mortality.

In addition, an ML model was constructed to predict all-cause hospital mortality in patients with PI using a binary classification approach. First, the correlation between variables was assessed using a Pearson correlation test, and those with coefficients >0.5 were excluded to reduce multicollinearity. Continuous variables were standardized and categorical variables were encoded. Feature selection was performed using LASSO regression and 3 ML algorithms, and the top 10 features were identified. These features were then tested again for Pearson correlation and VIFs before being incorporated into 11 common ML algorithms (eg, light gradient boosting machine [LightGBM], random forest, and extreme gradient boosting). The dataset was divided into a training and internal validation cohort in a 7:3 ratio. Model training and evaluation were performed using 10-fold cross-validation (repeatedcv, repeats =1) to reduce the risk of overfitting. Hyperparameter tuning was conducted with a random search strategy in R using the trainControl function, and the optimal parameters are shown in Table S1 in Multimedia Appendix 1. Model performance was compared with SOFA and SAPS II scores using ROC curves and multiple metrics, including AUC, accuracy, sensitivity, specificity, *F*_1_-score, geometric mean, precision, and recall. For the best-performing ML models, Shapley Additive Explanations (SHAP) were used to assess feature importance and generate partial dependence plots. Finally, a web-based prediction platform was developed based on the best-performing models to improve clinical accessibility.

All statistical analyses were performed using SPSS (version 23.0; IBM Corp), R (version 4.3.2), and Python (version 3.11.1; Python Software Foundation).

### Ethical Considerations

This study was a retrospective observational cohort study using the publicly available MIMIC-IV database (version 2.2), which contains deidentified patient data. The IRB of BIDMC waived the requirement for informed consent, as all data in the MIMIC-IV database are deidentified. The use of the database for this research was approved by the IRB of BIDMC. To comply with relevant regulations, the author, HF, obtained a Collaborative Institutional Training Initiative certification (61863057) and the necessary permissions to access and use the MIMIC-IV database. All procedures involving human subjects in this study adhered to the ethical standards of the institutional and national research councils as well as the 1964 Declaration of Helsinki and its subsequent amendments or equivalent ethical standards. As this study used the publicly available MIMIC-IV data, no participants were directly involved and no compensation was provided.

## Results

### Baseline Characteristics

**Figure 1.**
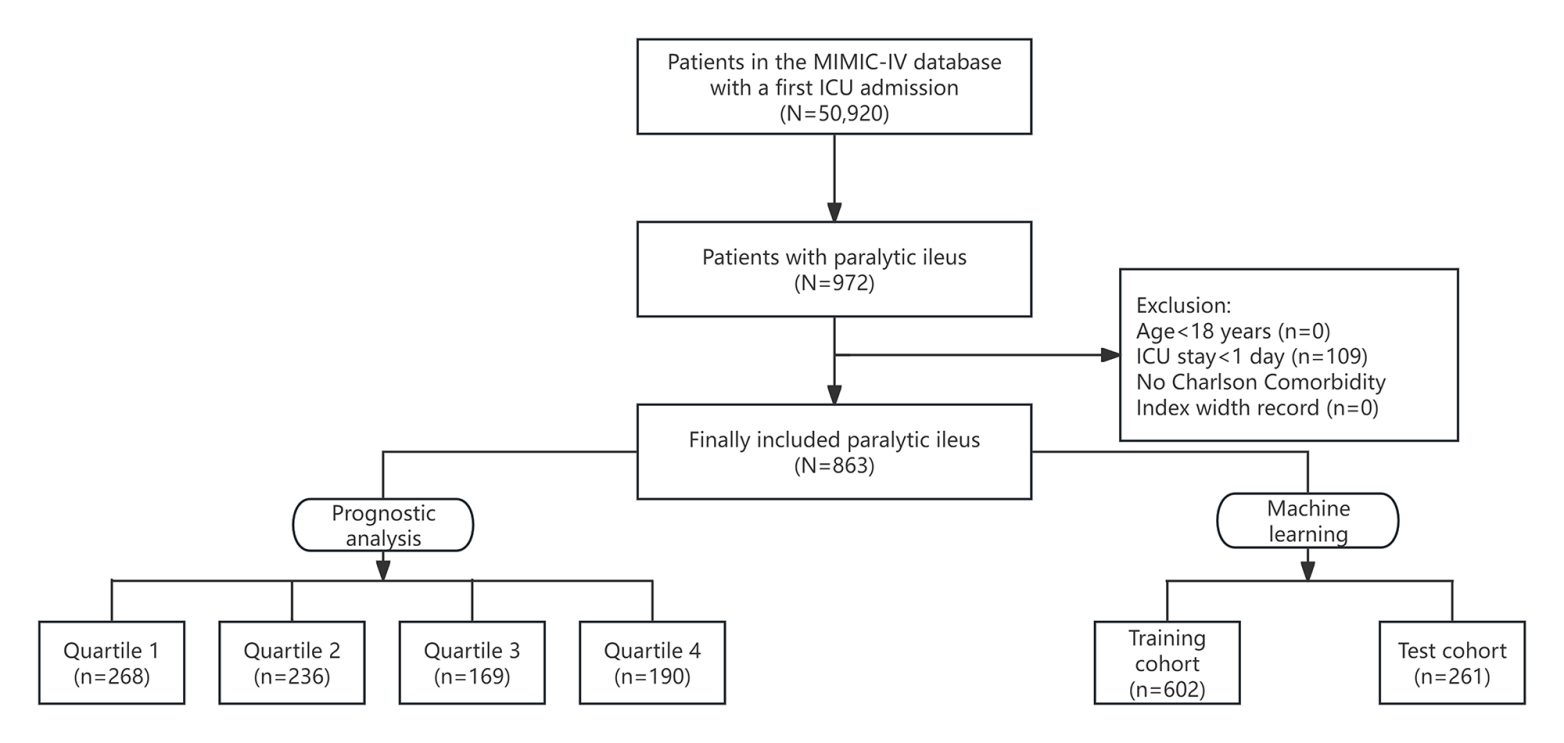
Flowchart of patient selection for the study cohort. ICU: intensive care unit; MIMIC-IV: Medical Information Mart for Intensive Care IV.

After screening the data of patients with PI in the MIMIC IV database, 863 patients who met the inclusion criteria were included in this study and divided into 4 groups according to the quartiles of CCI (Figure 1). Table 1 shows the baseline characteristics of critically ill patients with PI stratified by CCI quartiles. Patients were divided into 4 groups based on their CCI level on admission (quartile 1: 0‐3; quartile 2: 3‐5; quartile 3: 5‐7; and quartile 4: 7‐14). The median CCI values for each quartile in each group were 2 (IQR 1‐3), 4.5 (IQR 4‐5), 6 (IQR 6‐7), and 9 (IQR 8‐10), respectively. The median age of the enrolled patients was 65.4 (IQR 54.6‐75.5) years, of which 575 (66.6%) were male. The median CCI for all participants was 5 (IQR 3‐7). Compared to the lower group, patients in the highest CCI quartile generally had higher age, RDW, anion gap, BUN, creatinine, SAPS II, OASIS, APS III, SOFA, lower MBP, heart rate, hemoglobin, higher CHF, renal disease, malignant cancer, diabetes prevalence, and lower hypertension prevalence. Hospital mortality, ICU mortality, 28-day mortality, and 90-day mortality were 157 (18.2%), 76 (9%), 171 (19.8%), and 233 (27%), respectively. As the Q4 group was better associated with all-cause mortality, the differences between Q4 and Q1-Q3 were further compared. The analysis showed that different grouping methods gave similar results (Table S2 in Multimedia Appendix 1).

**Table 1.** Characteristics and outcomes of intensive care unit (ICU) patients with paralytic ileus categorized by Charlson Comorbidity Index (CCI) levels^a^ (N=863).

Categories	Overall	Q1 (n=268)	Q2 (n=236)	Q3 (n=169)	Q4 (n=190)	*P* value
Sex (male), n (%)	575 (66.6)	176 (65.7)	160 (67.8)	109 (64.5)	130 (68.4)	.83
Age (y), median (IQR)	65.4 (54.6‐75.5)	52.8 (42‐59.3)	67.4 (58‐76.5)	71.4 (62.4‐80.3)	73.6 (66.7‐81.7)	<.001
Race, n (%)						.64
White	576 (66.7)	175 (65.3)	153 (64.8)	115 (68.0)	133 (70)	
Others	287 (33.3)	93 (35)	83 (35)	54 (32)	57 (30)	
Comorbidities, n (%)						
CHF^b^	220 (25.5)	17 (6)	53 (23)	68 (40)	82 (43)	<.001
Renal disease	194 (22.5)	4 (2)	26 (11)	59 (35)	105 (55.3)	<.001
Malignant cancer	161 (18.7)	14 (5)	28 (12)	33 (20)	86 (45)	<.001
Sepsis	282 (32.7)	76 (28)	70 (30)	65 (39)	71 (37)	.052
Diabetes	226 (26.2)	34 (13)	55 (23)	45 (27)	92 (48)	<.001
Hypertension	355 (41.1)	97 (36)	133 (56.4)	74 (44)	51 (27)	<.001
Vital signs, median (IQR)						
RR^c^ (breaths/min)	18.8 (16.4‐21.9)	19.0 (16.6‐21.9)	19.1 (16.1‐21.7)	19.1 (16.6‐23.0)	18.3 (16.4‐21.9)	.60
MBP^d^ (mmHg)	73.9 (68.6‐81.8)	77.4 (71.3‐84.7)	73.9 (68.5‐82.2)	73.3 (68.5‐79.0)	72.2 (66.5‐78.1)	<.001
Heart rate (bpm)	89.2 (79.2‐103)	93.0 (81.3‐106)	88.1 (79.1‐101)	88.6 (77.8‐102)	86.4 (76.5‐99.0)	.001
SpO_2_^e^(%)	97.0 (95.5‐98.4)	97.2 (95.8‐98.5)	96.7 (95.1‐98.2)	96.8 (95.2‐98.3)	97.3 (95.7‐98.4)	.09
Laboratory test, median (IQR)						
WBC^f^ (K/µL)	12.8 (9.15‐18.0)	13.2 (9.20‐18.2)	12.4 (8.90‐18.2)	13.4 (9.20‐19.7)	12.0 (8.98‐15.7)	.28
RDW^g^ (%)	15.0 (13.9‐16.6)	14.5 (13.6‐15.7)	15.1 (13.8‐16.5)	15.6 (14.1‐17.0)	15.4 (14.4‐17.1)	<.001
Platelet (K/µL)	176 (113‐235)	190 (126‐241)	158 (99.5‐225)	171 (119‐233)	175 (119‐234)	.15
Hemoglobin (g/dL)	9.60 (8.40‐11.0)	10.0 (8.60‐11.5)	9.50 (8.20‐11.0)	9.70 (8.30‐11.0)	9.20 (8.10‐10.4)	.001
Anion gap (mmol/L)	14.0 (12.0‐17.0)	14.0 (11.0‐16.0)	14.0 (11.0‐16.0)	14.0 (13.0‐17.0)	15.0 (12.0‐17.0)	.001
BUN^h^ (mg/dL)	21.0 (14.0‐34.0)	15.0 (11.0‐23.0)	22.0 (15.0‐34.1)	26.0 (18.0‐38.0)	30.0 (18.2‐40.8)	<.001
Creatinine (mg/dL)	1.10 (0.80‐1.60)	0.90 (0.60‐1.20)	1.10 (0.80‐1.50)	1.20 (0.90‐1.63)	1.45 (1.00‐1.81)	<.001
Calcium (mg/dL)	8.10 (7.70‐8.60)	8.00 (7.60‐8.50)	8.10 (7.72‐8.60)	8.20 (7.80‐8.60)	8.12 (7.70‐8.60)	.08
Chloride (mEq/L)	105 (101‐109)	105 (101‐109)	105 (101‐109)	104 (99.0‐108)	105 (101‐108)	.22
Sodium (mEq/L)	138 (135‐141)	138 (136‐141)	138 (135‐141)	138 (135‐141)	139 (135‐141)	.71
Potassium (mEq/L)	4.20 (3.80‐4.50)	4.10 (3.70‐4.40)	4.20 (3.70‐4.60)	4.20 (3.80‐4.60)	4.20 (3.80‐4.60)	.09
Severity scores, median (IQR)						
SAPS II^i^	37.0 (30.0‐47.0)	30.0 (22.0‐38.0)	37.0 (31.0‐45.0)	40.0 (33.0‐51.0)	43.0 (37.0‐54.0)	<.001
OASIS^j^	33.0 (27.0‐39.5)	31.0 (25.0‐38.0)	33.0 (29.0‐39.0)	34.0 (27.0‐40.0)	34.0 (28.0‐41.0)	.001
APS III^k^	47.0 (35.0‐63.5)	41.0 (30.0‐59.0)	48.0 (34.0‐63.0)	50.0 (41.0‐67.0)	51.0 (38.0‐69.6)	<.001
SOFA^l^	2.00 (1.00‐5.00)	2.00 (1.00‐4.00)	2.00 (1.00‐5.00)	3.00 (1.00‐5.00)	3.00 (1.00‐5.00)	<.001
CCI	5.00 (3.00‐7.00)	2.00 (1.00‐3.00)	4.50 (4.00‐5.00)	6.00 (6.00‐7.00)	9.00 (8.00‐10.0)	<.001
Medication, n (%)						
Ondansetron	475 (55.0)	154 (57.5)	124 (52.5)	92 (54)	105 (55.3)	.74
Neostigmine	101 (11.7)	30 (11)	34 (14)	23 (14)	14 (7)	.12
Events, median (IQR)						
Hospital LOS^m^ (d)	14.6 (9.72‐23.1)	14.4 (9.27‐24.0)	14.1 (9.32‐24.5)	15.5 (10.6‐22.7)	15.0 (10.5‐20.8)	.87
ICU LOS (d)	3.65 (1.94‐7.85)	3.30 (1.91‐7.74)	3.92 (1.91‐8.79)	4.03 (2.08‐7.51)	3.30 (1.96‐7.61)	.58
Mortality, n (%)						
Hospital	157 (18.2)	26 (10)	46 (20)	34 (20)	51 (27)	<.001
ICU	76 (9)	17 (6)	22 (9)	17 (10)	20 (10)	.37
28 days	171 (19.8)	23 (9)	44 (19)	40 (24)	64 (34)	<.001
90 dayss	233 (27.0)	32 (12)	61 (26)	54 (32)	86 (45)	<.001

a Continuous data are presented as median (IQR), whereas categorical data are presented as n (%), Q1 (0‐3), Q2 (3–5), Q3 (5–7), Q4 (7–14).

b CHF: congestive heart failure.

c RR: respiratory rate.

d MBP: mean blood pressure.

e SpO_2_: saturation of peripheral oxygen.

f WBC: white blood cell.

g RDW, red blood cell distribution width.

h BUN: blood urea nitrogen.

i SAPS II: Simplified Acute Physiological Score II.

j OASIS: Oxford Acute Severity of Illness Score.

k APS III: Acute Physiology Score III.

l SOFA: Sequential Organ Failure Assessment.

m LOS: length of stay.

Table 2 shows the differences in baseline characteristics between survivors and nonsurvivors during hospitalization. Patients in the nonsurvivor group were more likely to have higher RR, heart rate, RDW, anion gap, BUN, creatinine, SAPS II, OASIS, APS III, SOFA, CCI, higher prevalence of CHF, sepsis, lower prevalence of hypertension, lower SpO_2_, platelets, hemoglobin, calcium, lower ondansetron, and neostigmine use. CCI values were significantly higher in the nonsurvivors than in the survivors (6 vs 5; *P*<.001). Figure S1 in Multimedia Appendix 1 shows the distribution of CCIs stratified by mortality status for inpatient deaths, ICU deaths, 28-day deaths, and 90-day deaths (Figure S2 in Multimedia Appendix 1).

**Table 2.** Baseline characteristics of intensive care unit (ICU) survivors and nonsurvivors with paralytic ileus^a^ (N=863).

Categories	Overall	Survivor (n=706)	Nonsurvivor (n=157)	*P* value
Sex (male), n (%)	575 (66.6)	469 (66.4)	106 (67.5)	.87
Age (y), median (IQR)	65.4 (54.6‐75.5)	65.3 (54.1‐75.4)	66.1 (56.0‐75.9)	.29
Race, n (%)				1.00
White	576 (66.7)	471 (66.7)	105 (66.9)	
Others	287 (33.3)	235 (33.3)	52 (33)	
Comorbidities, n (%)				
CHF^b^	220 (25.5)	169 (23.9)	51 (33)	.03
Renal disease	194 (22.5)	149 (21.1)	45 (29)	.05
Malignant cancer	161 (18.7)	123 (17.4)	38 (24)	.06
Sepsis	282 (32.7)	187 (26.5)	95 (61)	<.001
Diabetes	226 (26.2)	188 (26.6)	38 (24)	.60
Hypertension	355 (41.1)	308 (43.6)	47 (30)	.002
Vital signs, median (IQR)				
RR^c^ (breaths/min)	18.8 (16.4‐21.9)	18.4 (16.3‐21.4)	20.5 (17.6‐23.9)	<.001
MBP^d^ (mmHg)	73.9 (68.6‐81.8)	74.6 (69.4‐82.4)	71.4 (66.4‐77.5)	<.001
Heart rate (bpm)	89.2 (79.2‐103)	88.6 (78.6‐102)	92.5 (80.9‐105)	.03
SpO_2_^e^ (%)	97.0 (95.5‐98.4)	97.2 (95.7‐98.4)	96.2 (94.8‐98.1)	<.001
Laboratory test, median (IQR)				
WBC^f^ (K/µL)	12.8 (9.15‐18.0)	12.6 (9.20‐17.7)	14.0 (8.90‐19.7)	.10
RDW^g^ (%)	15.0 (13.9‐16.6)	14.9 (13.8‐16.3)	16.4 (14.5‐17.6)	<.001
Platelet (K/µL)	176 (113‐235)	180 (121‐237)	147 (67.0‐227)	.001
Hemoglobin (g/dL)	9.60 (8.40‐11.0)	9.70 (8.40‐11.1)	9.11 (8.20‐11.0)	.04
Anion gap (mmol/L)	14.0 (12.0‐17.0)	14.0 (12.0‐16.0)	15.0 (13.0‐18.0)	<.001
BUN^h^ (mg/dL)	21.0 (14.0‐34.0)	20.0 (14.0‐31.0)	31.0 (18.0‐41.9)	<.001
Creatinine (mg/dL)	1.10 (0.80‐1.60)	1.00 (0.80‐1.50)	1.30 (0.90‐1.80)	<.001
Calcium (mg/dL)	8.10 (7.70‐8.60)	8.11 (7.70‐8.60)	8.00 (7.50‐8.50)	.04
Chloride (mEq/L)	105 (101‐109)	105 (101‐109)	104 (99.0‐109)	.16
Sodium (mEq/L)	138 (135‐141)	138 (136‐141)	138 (134‐141)	.14
Potassium (mEq/L)	4.20 (3.80‐4.50)	4.10 (3.73‐4.50)	4.20 (3.80‐4.70)	.23
Severity scores, median (IQR)				
SAPS II^i^	37.0 (30.0‐47.0)	35.0 (28.0‐43.0)	47.0 (37.0‐56.0)	<.001
OASIS^j^	33.0 (27.0‐39.5)	32.0 (27.0‐39.0)	36.0 (30.0‐44.0)	<.001
APS III^k^	47.0 (35.0‐63.5)	44.0 (33.0‐58.0)	68.0 (50.0‐77.4)	<.001
SOFA^l^	2.00 (1.00‐5.00)	2.00 (1.00‐4.00)	4.00 (2.00‐6.27)	<.001
CCI^m^	5.00 (3.00‐7.00)	5.00 (3.00‐7.00)	6.00 (4.00‐8.00)	<.001
Medication, n (%)				
Ondansetron	475 (55.0)	413 (58.5)	62 (40)	<.001
Neostigmine	101 (11.7)	96 (14)	5 (3)	<.001

a Continuous data are presented as median (IQR), whereas categorical data are presented as frequency (%).

b CHF: congestive heart failure.

c RR: respiratory rate.

d MBP: mean blood pressure.

e SpO_2_: saturation of peripheral oxygen.

f WBC: white blood cell.

g RDW: red cell distribution width.

h BUN: blood urea nitrogen.

i SAPS II: Simplified Acute Physiological Score II.

j OASIS: Oxford Acute Severity of Illness Score.

k APS III: Acute Physiology Score III.

l SOFA: Sequential Organ Failure Assessment.

m CCI: Charlson Comorbidity Index.

### Primary Outcomes

**Figure 2.**
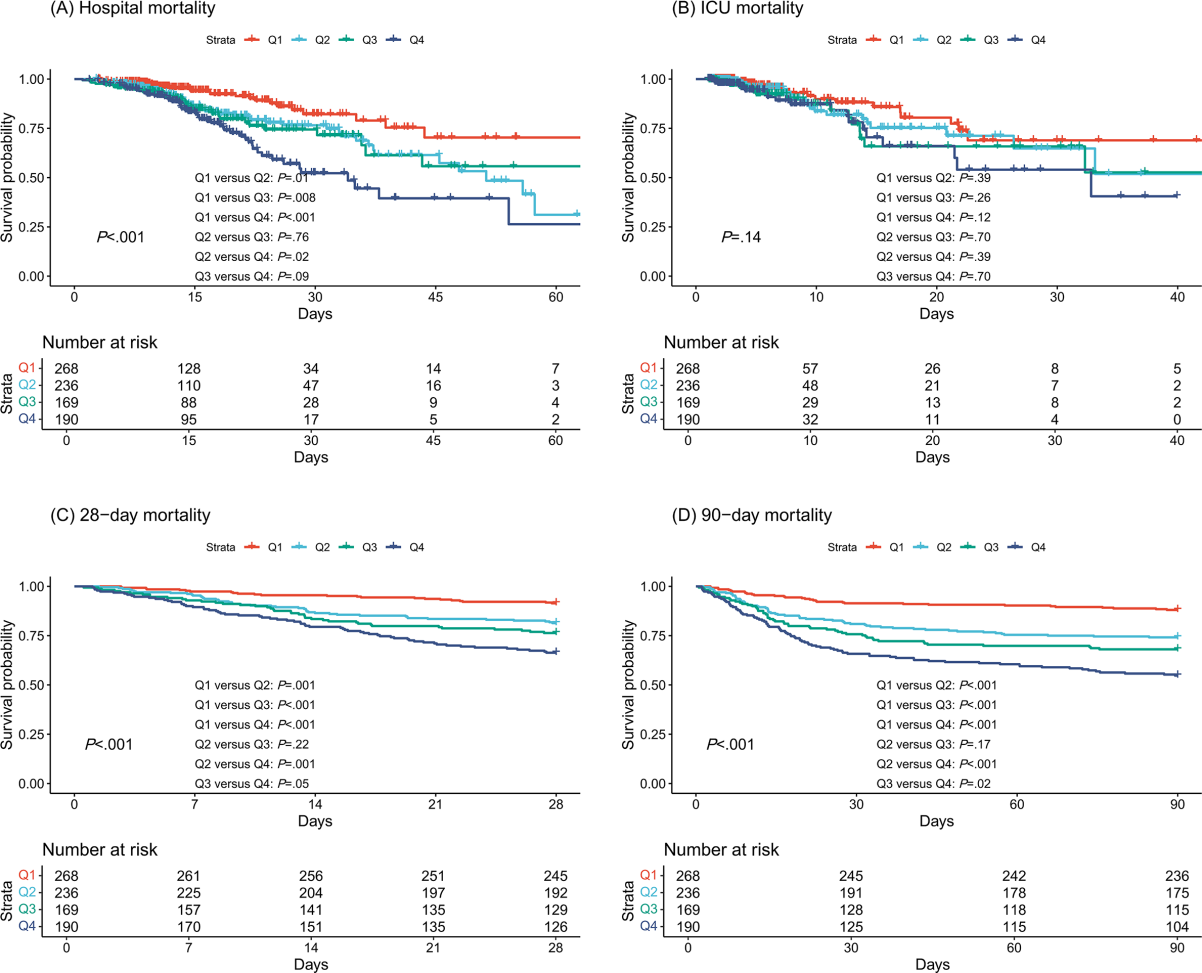
Kaplan-Meier survival curves for all-cause hospital, intensive care unit (ICU), 28-day, and 90-day mortality in ICU patients with paralytic ileus. Charlson Comorbidity Index quartiles were defined as Q1 (0‐3), Q2 (3–5), Q3 (5–7), and Q4 (7–14). Panels show cumulative probability of all-cause mortality at (A) hospital, (B) ICU, (C) 28-day, and (D) 90-day follow-up.

Kaplan-Meier survival analyses showed that patients with higher CCI scores had a significantly higher risk of hospital, 28-day, and 90-day all-cause mortality (Figure 2). ROC curve analyses assessed the clinical efficacy of the CCI (Figure S3 in Multimedia Appendix 1) and showed that the AUCs of the CCIs were all greater than 0.5, which to some extent discriminated patients who died in hospital, ICU, 28-day, and 90-day from those who did not (AUC for hospital deaths: 0.631, odds ratio [OR] 1.17, 95% CI 1.10‐1.24, *P*<.001; AUC for ICU deaths: 0.58, OR 1.10, 95% CI 1.02‐1.20, *P*=.02; 28-day death AUC: 0.678, OR 1.24, 95% CI 1.17‐1.32, *P*<.001; 90-day ICU death AUC: 0.687, OR 1.27, 95% CI 1.20‐1.34, *P*<.001). The optimal CCI cut-off for hospital, ICU, 28-day, and 90-day death was 4.5.

**Figure 3.**
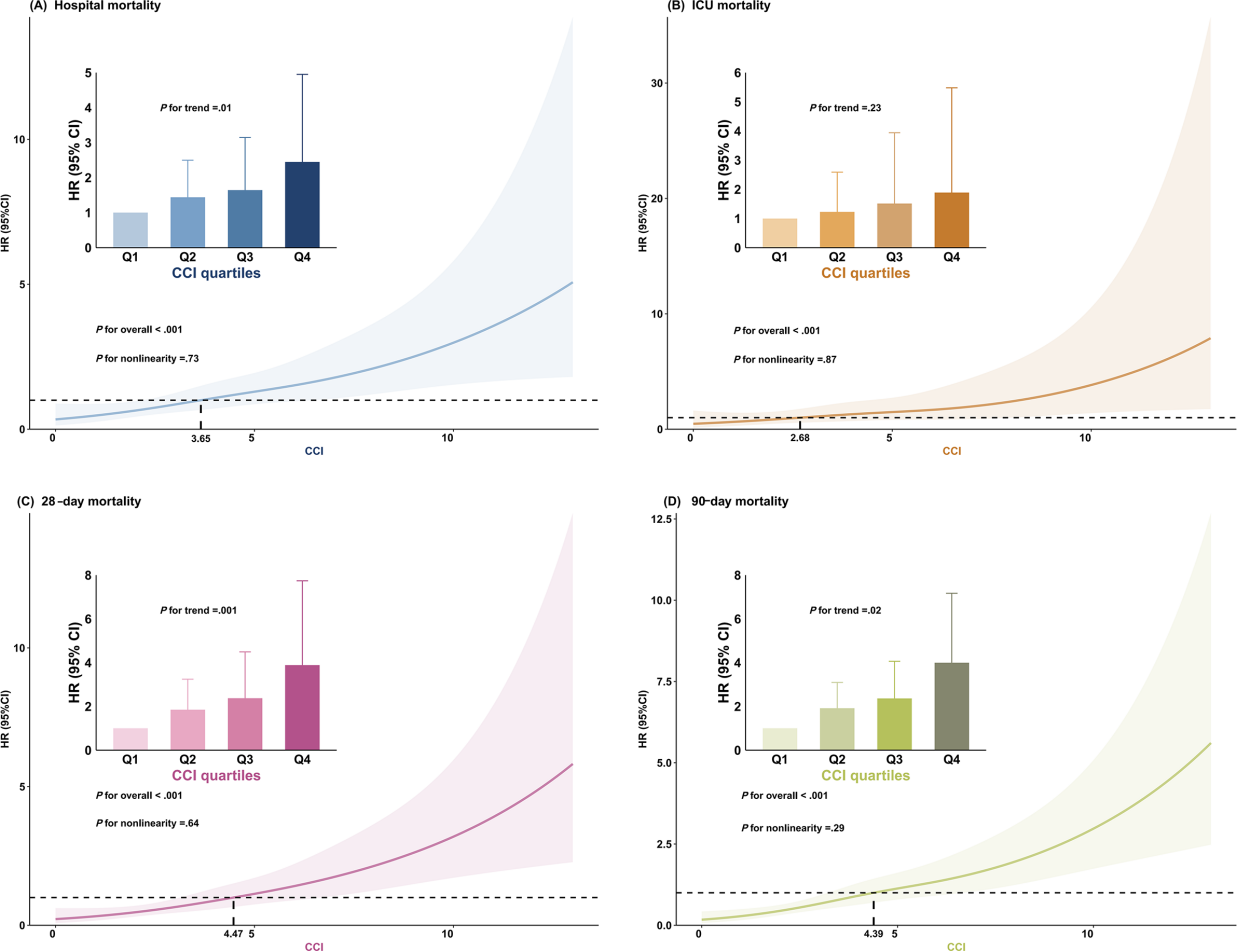
Hazard ratios (HRs) and restricted cubic spline curves for all-cause mortality from Charlson Comorbidity Index (CCI) after adjustment for confounders. All models were adjusted for multiple confounders, including sex, age, congestive heart failure (CHF), renal disease, sepsis, hypertension, diabetes, white blood cell count, red blood cell distribution width, anion gap, creatinine, and CCI. The bar graph shows the HRs for all-cause mortality between different CCI quartiles, with the CIs for each group. The first quartile of CCI is the reference group to which the other quartiles are compared. The central thick solid line represents the estimated HR after adjustment, the shaded area represents the CIs, and the horizontal dashed line represents the HR of 1.0. The vertical dashed line corresponds to the CCI value where the HR is 1.0. (**A**) Hospital mortality: the vertical dashed line corresponds to a CCI value of 3.65. (**B**) Intensive care unit (ICU) mortality: the vertical dashed line corresponds to a CCI value of 2.68. (**C**) 28-day mortality: the vertical dashed line corresponds to a CCI value of 4.47. (**D**) 90-day mortality: the vertical dashed line corresponds to a CCI value of 4.39.

Cox regression analysis was used to assess the association between CCI and in-hospital mortality. The results showed that when CCI was a continuous variable, CCI was a significant risk factor in the unadjusted model (hazard ratio [HR] 1.159, 95% CI 1.099‐1.222; *P*<.001), the partially adjusted model (HR 1.158, 95% CI 1.088‐1.233; *P*<.001), and the fully adjusted model (HR 1.204, 95% CI 1.099‐1.319; *P*<.001). When CCI was the ordinal variable, patients in higher quartiles of CCI were significantly associated with a higher risk of in-hospital death compared with participants in the lowest quartile in all 3 established Cox regression analysis models: unadjusted model (HR 3.124, 95% CI 1.945‐5.017; *P*<.001), partially adjusted model (HR 2.950, 95% CI 1.708‐5.097; *P*<.001), and fully adjusted model (HR 2.447, 95% CI 1.210‐4.951; *P*=.01) and showed an increasing trend with increasing CCI (Table 3 and Figure 3A). Similar results were observed in multivariate Cox regression analyses for 28-day mortality (Table 3 and Figure 3) and 90-day mortality (Table S3 in Multimedia Appendix 1; Figure 3), but less favorable results were observed in multivariate Cox regression analyses for CCI and ICU mortality (Table S3 in Multimedia Appendix 1; Figure 3). In addition, the use of an RCS regression model showed a linear increase in the risk of hospital, ICU, 28-day, and 90-day mortality with increasing CCI (nonlinear *P* values of .73, .87, .64, and .29, respectively; Figure 3).

**Table 3.** Cox proportional hazard ratios (HRs) for all-cause hospital and 28-day mortality in intensive care unit (ICU) patients with paralytic ileus.

Categories	Model 1^a^			Model 2^b^			Model 3^c^		
HR (95% CI)	*P* value	*P* for trend	HR (95% CI)	*P* value	*P* for trend	HR (95% CI)	*P* value	*P* for trend
Hospital mortality									
Continuous variable	1.159 (1.099‐1.222)	<.001		1.158 (1.088‐1.233)	<.001		1.204 (1.099‐1.319)	<.001	
Per unit quartile^d^	<.001		<.001		.01
Q1 (n=190)	Ref						Ref		
Q2 (n=162)	1.936 (1.196‐3.135)	.007		1.868 (1.119‐3.117)	.02		1.442 (0.832‐2.499)	.19	
Q3 (n=159)	2.091 (1.254‐3.487)	.004		2.003 (1.140‐3.518)	.02		1.643 (0.857‐3.149)	.14	
Q4 (n=170)	3.124 (1.945‐5.017)	<.001		2.950 (1.708‐5.097)	<.001		2.447 (1.210‐4.951)	.01	
28-day mortality									
Continuous variable	1.202 (1.143‐1.264)	<.001		1.237 (1.166‐1.311)	<.001		1.255 (1.154‐1.366)	<.001	
Per unit quartile^d^			<.001			<.001			<.001
Q1 (n=190)	Ref						Ref		
Q2 (n=162)	2.315 (1.398‐3.833)	.001		2.683 (1.567‐4.594)	<.001		1.849 (1.054‐3.245)	.03	
Q3 (n=159)	3.035 (1.817‐5.069)	<.001		3.660 (2.087‐6.427)	<.001		2.375 (1.256‐4.493)	.008	
Q4 (n=170)	4.563 (2.833‐7.349)	<.001		5.668 (3.267‐9.833)	<.001		3.891 (1.956‐7.740)	<.001	

a Model 1: unadjusted.

b Model 2: unadjusted.

c Model 3: adjusted for sex, age, congestive heart failure (CHF), renal disease, malignant cancer, sepsis, diabetes, hypertension, respiratory rate (RR), saturation of peripheral oxygen (SpO_2_), white blood cell (WBC), red blood cell distribution width (RDW), platelets, anion gap, blood urea nitrogen (BUN), creatinine, Simplified Acute Physiological Score II (SAPS II), Oxford Acute Severity of Illness Score (OASIS), Acute Physiology Score III (APS III), Sequential Organ Failure Assessment (SOFA), and Charlson Comorbidity Index (CCI).

d CCI: Q1 (0‐3), Q2 (3–5), Q3 (5–7), Q4 (7–14)

### Subgroup Analysis

**Figure 4.**
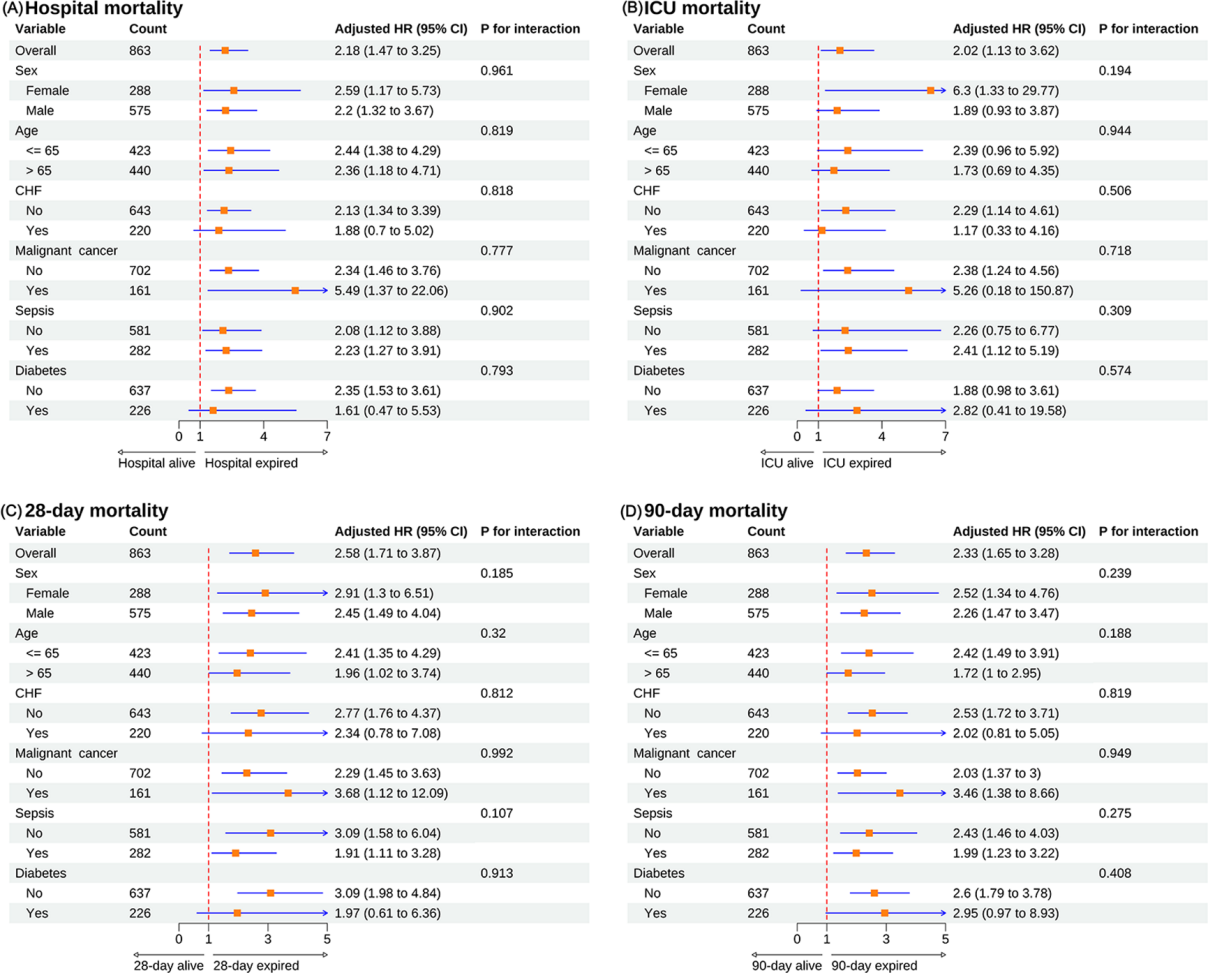
Subgroup analyses of the association between Charlson Comorbidity Index (CCI) and all-cause mortality in intensive care unit (ICU) patients with paralytic ileus. (A) Hospital mortality. (**B**) ICU mortality. (**C**) 28-day mortality. (**D**) 90-day mortality. CHF: congestive heart failure; HR: hazard ratio.

Subgroup analyses by sex, age, CHF, malignancy, sepsis, and diabetes showed no interaction (all interactions *P*>.05), that is, the association between CCI and the risk of all-cause hospital mortality did not change with changes in the stratification variables of sex, age, CHF, malignant cancer, sepsis, and diabetes levels (Figure 4).

### Sensitivity Analysis

Table S4 in Multimedia Appendix 1 shows that the association between CCI and mortality outcomes in patients with PI remained stable and similar after excluding patients who died within 3 days of ICU admission.

Table S5 in Multimedia Appendix 1 shows that the sample was divided into groups of ≤4.5 and >4.5 based on the optimal cut-off value for CCI. The results show that regardless of the grouping method used, the conclusions obtained are consistent with the results of the main analysis.

A further sensitivity analysis of the SOFA score for the 3 groups was performed. The analysis showed that for the different subgroups of SOFA scores, consistent associations were observed between CCI in the higher quartiles and mortality from PI, supporting the stability of the findings (Table S6 in Multimedia Appendix 1).

### Feature Preselection for ML Models

Baseline characteristics of patients in the training and test cohorts are shown in Table S7 in Multimedia Appendix 1. Figure S4 in Multimedia Appendix 1 shows the heatmaps of all variables for which correlations are provided. Because BUN and creatinine (*r*=0.69), SAPS II and APS III (*r*=0.69) or OASIS (*r*=0.68), OASIS and APS III (*r*=0.62), age and CCI (*r*=0.59), and chloride and sodium (*r*=0.66), BUN, SAPS II, OASIS, age, and chloride were excluded in the next step. In the training queue, the 3 ML algorithms with default parameters and the LASSO algorithm preselected the 10 features used to construct the model (Figure S5 in Multimedia Appendix 1), with CCI ranking in the top 10 in each algorithm. Importantly, there is no strong correlation or multicollinearity between the selected features (Figure S6 in Multimedia Appendix 1).

### ML Model Construction and Evaluation

**Figure 5.**
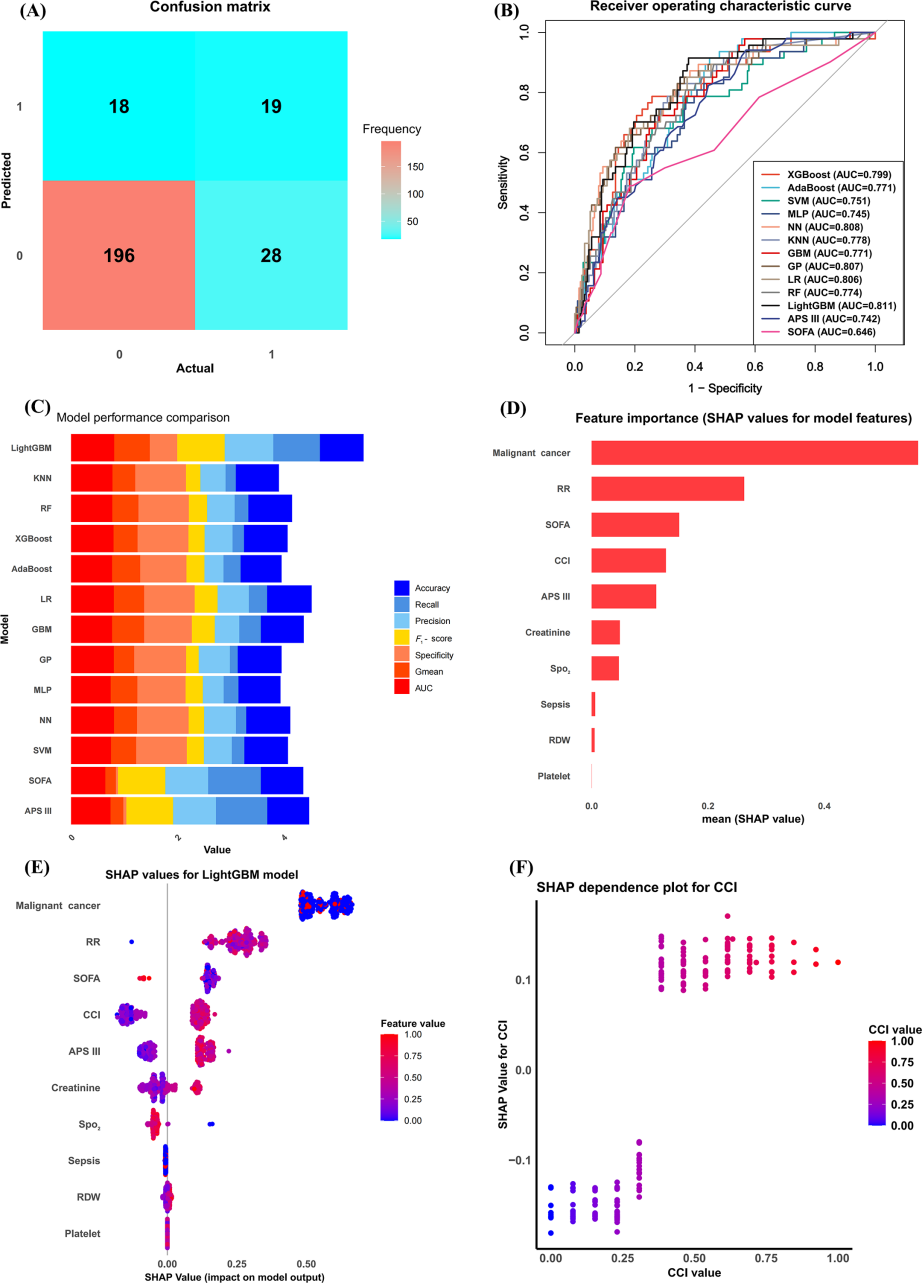
(A) Machine-learning model development, evaluation, and feature importance visualization. Confusion matrix of the light gradient boosting machine (LightGBM) model. (B) Receiver operating characteristic (ROC) curves of 11 machine-learning models. (C) Various evaluation metrics of 11 machine-learning models. (D) Feature importance plot of the LightGBM model. (E) Shapley Additive Explanations (SHAP) plot of the Charlson Comorbidity Index (CCI) feature. (F) SHAP dependence plot of the CCI. AdaBoost: adaptive boosting; APS III: Acute Physiology Score III; AUC: area under the curve; GP: Gaussian process; LR: logistic regression; KNN: k-nearest neighbor; MLP: multilayer perceptron; NN: neural network; RDW: red blood cell distribution width; RF: random forest; RR: respiratory rate; SOFA: Sequential Organ Failure Assessment; SPO_2_: peripheral capillary oxygen saturation; SVM: support vector machine; XGBoost: extreme gradient boosting.

The best hyperparameters were determined after inputting the selected features into the 11 ML models. Then, Figure 5A is the confusion matrix of the LightGBM model, and the ROC curves (Figure 5B) and other metrics (Figure 5C) were evaluated among all the ML models. LightGBM was considered the best model, outperforming other ML models including random forest and extreme gradient boosting model. This is because the internal validation results show that it has the highest AUC (0.811), *F*_1_-score (0.895), and geometric mean (0.670) and outperforms both the traditional SOFA score and the APS III score (Table 4).

**Table 4.** Performance evaluation of 11 machine learning models for predicting hospital mortality in intensive care unit (ICU) patients with paralytic ileus.

Model	Accuracy	Recall	Precision	*F*_1_-score	Specificity	Gmean	AUC
SVM^a^	0.824	0.234	0.524	0.324	0.953	0.472	0.751
NN^b^	0.831	0.191	0.600	0.290	0.972	0.431	0.808
MLP^c^	0.793	0.277	0.394	0.325	0.907	0.501	0.745
GP^d^	0.828	0.149	0.583	0.237	0.977	0.381	0.807
GBM^e^	0.808	0.404	0.463	0.432	0.897	0.602	0.771
LR^f^	0.839	0.340	0.593	0.432	0.949	0.568	0.806
AdaBoost^g^	0.774	0.319	0.357	0.337	0.874	0.528	0.771
XGBoost^h^	0.824	0.213	0.526	0.303	0.958	0.451	0.799
RF^i^	0.824	0.255	0.522	0.343	0.949	0.492	0.774
KNN^j^	0.816	0.191	0.474	0.273	0.953	0.427	0.778
LightGBM^k^	0.824	0.875	0.916	0.895	0.514	0.670	0.811
APS III^l^	0.787	0.966	0.806	0.879	0.059	0.238	0.742
SOFA^m^	0.802	0.99	0.807	0.889	0.039	0.197	0.646

a SVM: support vector machine.

b NN: neural network.

c MLP: multilayer perceptron.

d GP: Gaussian process.

e GBM: gradient boosting machine.

f LR: logistic regression.

g AdaBoost: adaptive boosting.

h XGBoost: extreme gradient boosting.

i RF: random forest.

j KNN: k-nearest neighbor.

k LightGBM: light gradient boosting machine.

l APS III: Acute Physiological Score III.

m SOFA: Sequential Organ Failure Assessment.

### Visualization of Feature Importance

The corresponding SHAP values for each feature in the internal validation cohort of the LightGBM model were calculated and ranked (Figures 5D and 5E), and CCI ranked third in significance compared to the other predictor variables. The SHAP dependence plot of the CCI (Figure 5F) showed that increased CCI was associated with an increased risk of hospital death in patients with PI.

### Web-Based Forecasting Platform

To improve the usability of the LightGBM model, it has been embedded in a user-friendly website for external users and practitioners to validate or make predictions [31]. This prototype website allows users to input clinical variables such as APS III, SOFA, Charlson Comorbidity Index, and laboratory parameters (platelet, RDW, creatinine, and others) to estimate mortality risk in patients with PI. It is intended solely for research demonstration, does not replace clinical judgment, and does not store any protected health information. For example, Figure S7A in Multimedia Appendix 1 illustrates the case of a patient with PI aged 48 years with the characteristics shown. The final output probability of hospital all-cause mortality is 0.68, indicating a high risk status. This website also allows you to view and download this patient’s SHAP force plot, as shown in Figure S7B in Multimedia Appendix 1.

## Discussion

### Principal Findings

This study analyzed the association between CCI and clinical outcomes in a US cohort of critically ill patients with PI, with the following key findings:

1. Higher levels of CCI were significantly associated with hospital, 28-day, and 90-day all-cause mortality in patients with PI. Even after correction for confounders, CCI remained strongly correlated with these mortality indicators, with a trend towards a linear relationship. Our study suggests that the critical value of 4.5 for CCI levels provides a potential reference for mortality risk stratification, helping clinicians to identify high-risk patients who require closer monitoring.
2. A LightGBM model was developed combining CCI and other clinical variables beyond the traditional critical illness score, and the results showed that CCI had a higher weight and contribution in the model. Overall, this study highlights the critical role of CCI in risk stratification of mortality in critically ill patients with PI, helping to optimize clinical management strategies.

CCI affects the prognosis of patients with PI through several interrelated biological mechanisms. First, CCI reflects a patient’s chronic disease burden, including conditions such as diabetes, neurological disorders, and malignant cancer, all of which increase all-cause mortality [5,6,8]. Chronic disease states are associated with persistent low-grade systemic inflammation, which can lead to endothelial dysfunction, oxidative stress, and vasoactive imbalances, which in turn exacerbate gut microcirculatory disturbances [32-34]. In addition, patients with high CCI often have suppressed immune function, as evidenced by decreased monocyte human leukocyte antigen–DR isotype expression, T-cell dysfunction, and prolonged immune paralysis, making them more susceptible to infectious complications [35,36]. The development of infection and sepsis can further exacerbate intestinal dysbiosis [37], leading to a vicious cycle of gut flora translocation and toxin release [38]. Dysbiosis may also affect the function of the enteric nervous system through abnormalities in short-chain fatty acid metabolism, thereby prolonging the course of PI [39-41]. In addition, patients with a higher CCI usually have a poor nutritional status, as evidenced by hypoproteinemia, muscle wasting, and inadequate metabolic reserve [42-44]. These factors may lead to reduced contractile function of the intestinal smooth muscle, delaying recovery of bowel function in PI and increasing the risk of death [45,46]. Taken together, CCI affects several key pathophysiological aspects of patients with PI, including chronic inflammation, immunosuppression, impaired intestinal barrier function, and malnutrition. The interaction of these mechanisms may explain why CCI is an important predictor of mortality in patients with PI and emphasizes the need for early detection and targeted intervention.

This study found that higher CCI was significantly associated with hospital, 28-day, and 90-day all-cause mortality in patients with PI, highlighting the importance of CCI in predicting the prognosis of critically ill patients. Patients with PI are often accompanied by a variety of comorbidities, such as diabetes mellitus and malignant cancer [5,8], and CCI, as a standardized measure of comorbidity burden, is effective in reflecting the systemic health status of the patient and in revealing the potential impact of comorbidities [9,10]. An international multicenter and subgroup bias assessment study showed that the risk of death in elderly critically ill patients increased with elevated CCI, emphasizing the profound prognostic impact of comorbidity accumulation [11]. In addition, this study also found that the critical value of CCI was 4.5 for hospital death, 28-day death, and 90-day death, a finding that further supports the important role of CCI in the prognostic assessment of patients with PI. There is no literature on the clinical value of CCI as a prognostic assessment tool for patients with PI, and this study is the first to explore and present this important finding. Compared with a single physiological parameter or laboratory index, CCI can provide more comprehensive information to the clinic by comprehensively assessing multidimensional health status, which may help in fine risk stratification and individualized treatment.

However, Cox regression analyses showed that patients with elevated CCI scores were associated with suboptimal ICU all-cause mortality outcomes after adjustment for confounders, while Kaplan-Meier survival analyses indicated that higher CCI scores were not significantly correlated with ICU mortality in patients with PI. This may be explained by the predominant role of acute care factors, such as the extent of organ failure, the intensity of systemic inflammation, and the severity of infection, which often outweigh the prognostic contribution of chronic disease burden in critically ill patients. For instance, prospective studies have demonstrated that inflammatory markers are associated with delirium, coma, and increased mortality risk [47], and multicenter cohort studies have further shown that inflammation and organ dysfunction are key determinants of 90-day mortality in acute respiratory distress syndrome [48]. In addition, intensive interventions commonly administered in the ICU, including mechanical ventilation, hemodynamic support, and renal replacement therapy, may mitigate the short-term impact of chronic comorbidities on mortality outcomes [49-51]. Thus, although the CCI remains a valuable tool for assessing chronic disease burden and predicting outcomes in hospitalized patients, its prognostic effect in ICU settings may be attenuated by acute care factors or interventions. Moreover, the relatively small sample size and low ICU mortality rate in this study may have limited statistical power. Future research should expand sample size and incorporate additional acute clinical indicators (eg, inflammatory markers and organ dysfunction scores) to further clarify the association between CCI and ICU mortality.

RCS regression analysis in this study showed that the level of CCI was significantly and linearly related to hospital mortality, ICU mortality, and 28- and 90-day mortality. Specifically, the threshold for hospital mortality was 3.65, the threshold for ICU mortality was 2.68, and the thresholds for 28-day and 90-day mortality were 4.47 and 4.39, respectively. These results suggest that lower CCI values (eg, thresholds for hospital and ICU mortality) indicate that the acute course of illness may be the primary risk factor for death, whereas higher thresholds reflect the importance of chronic disease burden in long-term prognosis. Chronic diseases, such as diabetes and malignant cancer, may have little impact on patient survival in the short term, but over time they progressively affect the patient’s immune system, organ function, and overall health [52,53], and may increase the risk of patient death in acute conditions, such as PI. Thus, the impact of chronic disease burden on prognosis was more pronounced during the 28- and 90-day observation periods, and long-term patient survival was closely linked to effective management of comorbidities. The results highlight the need to closely monitor and adjust patients’ CCI levels in clinical practice to optimize their short- and long-term prognosis.

Subgroup analyses showed no significant differences between subgroups (sex, age, CHF, malignant cancer, sepsis, and diabetes) in the effect of CCI on hospital, ICU, and 28-day and 90-day mortality. CCI levels have been shown to be strongly associated with poor prognosis in patients with CHF and malignant cancer [54,55]. Gadre et al [56] concluded that CCI ≥2 is a high-risk factor for readmission after sepsis hospitalization and may serve as an important indicator of poor prognosis in sepsis. These findings highlight the potential of CCI in a wide range of patient populations. Notably, in the sensitivity analysis of this study, the association between CCI and mortality outcomes in patients with PI remained stable and similar after excluding patients who died within 3 days of ICU admission. In addition, based on the optimal cut-off value of 4.5 for CCI grouping, the results obtained were consistent with the results of the primary analysis, further validating the reliability of CCI for predicting mortality. Finally, 3-group sensitivity analyses on SOFA scores were performed, and consistent associations between CCI and mortality were observed in the higher quartiles, supporting the stability and consistency of the findings. These sensitivity analyses demonstrate the strong robustness of CCI in predicting mortality in patients with PI under different subgroups and assumptions.

This study successfully developed and validated an ML model incorporating CCI for predicting hospital mortality in patients with PI. A total of 10 features were selected by a combination of LASSO and other ML algorithms, of which CCI ranked high in all algorithms, indicating the importance of CCI in predicting hospital mortality. This is similar to previous studies, many of which have demonstrated the importance of CCI in predicting mortality in a variety of patient populations, particularly critically ill patients [11,54,55]. This result is also consistent with the results of our previous Cox regression analysis. The performance of the ML model, SOFA [57] and APS III score [58], was evaluated by several metrics such as confusion matrix, ROC curve, and *F*_1_-score. It was found that the LightGBM algorithm outperformed other ML models, traditional SOFA and APS III scoring systems, achieving an AUC of 0.811 and an *F*_1_-score of 0.895, demonstrating its excellent predictive ability. These results are consistent with a growing body of literature showing that LightGBM has significant advantages in clinical prediction tasks, for example, Chen et al [20] concluded that LightGBM models outperform ML models such as random forest and support vector machines in predicting mortality in critically ill patients with atrial fibrillation. Feature importance analysis further highlighted the critical role of CCI in predicting mortality. SHAP value analysis, an interpretable ML technique, revealed that increasing CCI was associated with a higher risk of death in patients with PI, a finding consistent with the known association between comorbidities and patient prognosis [11,59]. The biased dependency plot also confirmed that increasing CCI values were associated with an increased risk of all-cause hospital mortality, further confirming its clinical relevance. Interpretable ML provides a more transparent model decision process and helps clinicians better understand the impact of features on predicted outcomes [60,61]. Razo et al [23] constructed a predictive model using deep learning and obtained an AUC of 0.887. However, because the predictive goal of this model is different from that of our study, it is not possible to directly compare the performance of the 2 models.

### Strengths and Limitations

This study has several important strengths. First, it provides validation that elevated CCI is an independent risk factor for short- and long-term mortality in critically ill patients with PI in a large US cohort. While prior studies have applied ML approaches to predict mortality in this population using the MIMIC database [23], our work extends these efforts by systematically evaluating the prognostic value of comorbidity burden as captured by the CCI. Second, this study methodologically advances by integrating CCI into a LightGBM-based ML framework and combining it with SHAP interpretability analysis. Finally, in practical terms, this web-based platform can be applied at the time of ICU admission to rapidly identify patients with PI at high risk of mortality, thereby supporting early triage, intensified monitoring, and timely interventions. By providing individualized risk estimates together with SHAP-based explanations, the tool not only enhances transparency of the prediction process but also facilitates communication between clinicians and patients’ families. Importantly, the model is designed to complement, rather than replace, physician judgment, and thus may serve as a practical decision-support tool within routine ICU workflows.

However, this study has some limitations. First, although a higher CCI was significantly associated with hospital, 28-day, and 90-day all-cause mortality in patients with PI, as a composite scoring tool, CCI still did not cover all clinical factors that may affect prognosis, such as B-type natriuretic peptide level, etiology of PI, individual patient differences, nutritional status, and cause of death. These factors not included in the analysis may have a significant impact on the risk of death, thus introducing a potential confounding bias. Second, given the retrospective design of this study, we could only identify associations between variables and not infer causality. Although there was a significant association between CCI and all-cause mortality, this does not mean that CCI was the direct cause of death. Third, CCI is mainly based on patients’ past medical history and does not take into account dynamic changes during the course of the disease, which may lead to limitations in capturing the complexity of the disease and the comprehensiveness of prognostic assessment. Fourth, although the ML-based LightGBM model performed well in internal validation, the absence of external validation using independent datasets (eg, eICU or non-US cohorts) limits its generalizability to broader populations. Fifth, given the multiple disease states and comorbidities of patients with PI, the model may be overly dependent on certain specific variables, ignoring complex interactions that may affect prognosis. Although multiple characteristics were included, the interactions between disease progression and comorbidities may not have been adequately considered. Future studies should improve the modeling of interactions between variables and dynamic factors to improve the accuracy of prognosis prediction. Sixth, although mortality was used as the primary end point in this study, considering the complexity of the prognosis of patients with PI, a single mortality indicator may not be able to comprehensively assess the health status and quality of life of patients. Therefore, future studies should explore more dimensional prognostic indicators, such as functional recovery, quality of life, and long-term survival, to provide a more comprehensive prognostic assessment. In conclusion, although CCI and ML-based models show promise in predicting mortality in critically ill patients with PI, their limitations need to be fully recognized in practical applications and combined with external validation data and dynamic clinical information to improve their clinical use and accuracy.

### Conclusions

This study demonstrated that higher CCI levels were strongly associated with hospital, 28-day, and 90-day all-cause mortality in patients with PI and were valuable in mortality risk stratification. Monitoring CCI levels in patients with PI in the ICU may be useful for early identification of high-risk patients and optimization of treatment strategies.

## Supplementary material

10.2196/76003 Multimedia Appendix 1 Hyperparameters and statistical analyses.

## Abbreviations

APS III: Acute Physiology Score III
AUC: area under the curve
BIDMC: Beth Israel Deaconess Medical Center
BUN: blood urea nitrogen
CCI: Charlson Comorbidity Index
CHF: congestive heart failure
HR: hazard ratio
ICD: International Classification of Diseases
ICU: intensive care unit
IRB: institutional review board
LASSO: least absolute shrinkage and selection operator
LightGBM: light gradient boosting machine
MBP: mean blood pressure
MIMIC: Medical Information Mart for Intensive Care
ML: machine learning
OASIS: Oxford Acute Severity of Illness Score
OR: odds ratio
PI: paralytic ileus
RCS: restricted cubic spline
RDW: red blood cell distribution width
ROC: receiver operating characteristic
RR: respiratory rate
SAPS II: Simplified Acute Physiology Score II
SHAP: Shapley Additive Explanations
SOFA: Sequential Organ Failure Assessment
SpO_2_: saturation of peripheral oxygen
VIF: variance inflation factor
WBC: white blood cell
