# Supplementary material for: Prognostic Value of the Charlson Comorbidity Index for Mortality and Machine Learning–Based Prediction in Critically Ill Patients with Paralytic Ileus: Retrospective Cohort Study
Source: JMIR Med Inform. 2025 Oct 16;13:e76003. doi: 10.2196/76003 (PMC12554354; doi:10.2196/76003)
Supplement: Multimedia Appendix 1 [file medinform-v13-e76003-s001.docx]

**Supplementary materials**

[Table S1. Best Hyperparameters for Various Machine Learning Models 2](#_Toc4298)

[Table S2. Characteristics and outcomes of participants categorized by Charlson Comorbidity Index 3](#_Toc7322)

[Table S3. Cox proportional hazard ratios (HRs) for all-cause ICU and 90-day mortality in patients with paralytic ileus 5](#_Toc20689)

[Table S4. Association of Charlson Comorbidity Index and all-cause mortality outcomes after excluding patients who died within three days of ICU admission 7](#_Toc17763)

[Table S5. Association of Charlson Comorbidity Index and all-cause mortality outcomes after stratification by CCI threshold (≤ 4.5 vs. > 4.5) 8](#_Toc1925)

[Table S6. Sensitivity analysis for the relationships between Charlson Comorbidity Index and all-cause mortality outcomes stratified by SOFA (≤1 vs. 2-4 vs. >4). 9](#_Toc31588)

[Table S7. Baseline characteristics of ICU patients with paralytic ileus in the training and test cohorts for machine learning model development. 11](#_Toc12023)

[Figure S1. Missing Value Ratio Plot and VIF Values for Independent Variables. 13](#_Toc12574)

[Figure S2. Distribution of the Charlson Comorbidity Index stratified by mortality status for Hospital, ICU, 28-day, and 90-day 14](#_Toc12574)

[Figure S3. ROC Analysis of Charlson Comorbidity Index for Predicting Mortality: Hospital, ICU, 28-day, 90-day 15](#_Toc13495)

[Figure S4. Correlation heatmap of all included variables 16](#_Toc10673)

[Figure S5. Feature selection for predicting hospital all-cause mortality after ICU admission in patients with paralytic ileus 17](#_Toc12712)

[Figure S6. Pearson’s correlation test (A) and variance inflation factor test (B) for selected features 18](#_Toc29317)

[Figure S7. Online platform for the light gradient boosting machine model (A) and SHAP Force Plot (B) 19](#_Toc15703)

| **Supplementary Table S1.** Best Hyperparameters for Various Machine Learning Models | |
| --- | --- |
| Model | Best Parameters |
| **SVM** | sigma = 0.08555629, C = 0.3459087 |
| **NN** | size = 14, decay = 2.262113 |
| **MLP** | size = 3 |
| **GP** | sigma = 0.02672977 |
| **GBM** | n.trees = 228, interaction.depth = 4, shrinkage = 0.06265188, n.minobsinnode = 7 |
| **LR** | not applicable |
| **AdaBoost** | mfinal = 51, maxdepth = 18, coeflearn = Freund |
| **XGBoost** | nrounds = 118, max_depth = 3, eta = 0.1742067, gamma = 7.584595, colsample_bytree = 0.3609779, min_child_weight = 1, subsample = 0.8592921 |
| **KNN** | kmax = 133, distance = 1.654305, kernel = cos |
| **RF** | mtry = 3 |
| **LightGBM** | objective = binary, metric = auc, boosting_type = gbdt, num_leaves = 70, learning_rate = 0.001, nrounds = 1000, feature_fraction = 0.5, bagging_fraction = 0.5, max_depth = 6, min_data_in_leaf = 70, lambda_l1 = 0.3, lambda_l2 = 0.3, is_unbalance = TRUE |
| Abbreviations: **SVM**, Support Vector Machine; **NN**, Neural Network; **MLP**, Multilayer Perceptron; **GP**, Gaussian Process; **GBM**, Gradient Boosting Machine; **LR**, Logistic Regression; **XGBoost**, Extreme Gradient Boosting; **KNN**, K-Nearest Neighbors; **RF**, Random Forest; **LightGBM**, Light Gradient Boosting Machine | |

**Supplementary Table S2.** Characteristics and outcomes of participants categorized by Charlson Comorbidity Index ^a^

| Categories | Overall (N=863 ) | Q1-Q3 (N=673) | Q4 (N=190) | *P*-value |
| --- | --- | --- | --- | --- |
| Sex: Male, n% | 575 (66.6) | 445 (66.1) | 130 (68.4) | .61 |
| Age (years) | 65.4(54.6-75.5) | 61.9(52.0-72.4) | 73.6(66.7-81.7) | <.001 |
| **Race, n%** |  |  |  | .32 |
| White | 576 (66.7) | 443 (65.8) | 133 (70.0) |  |
| Others | 287 (33.3) | 230 (34.2) | 57 (30) |  |
| **Comorbidities, n (%)** |  | | | |
| CHF | 220 (25.5) | 138 (20.5) | 82 (43) | <.001 |
| Renal disease | 194 (22.5) | 89 (13) | 105 (55.3) | <.001 |
| Malignant cancer | 161 (18.7) | 75 (11) | 86 (45) | <.001 |
| Sepsis | 282 (32.7) | 211 (31.4) | 71 (37) | .14 |
| Diabetes | 226 (26.2) | 134 (19.9) | 92 (48) | <.001 |
| Hypertension | 355 (41.1) | 304 (45.2) | 51 (27) | <.001 |
| **Vital signs** |  | | | |
| RR (breaths/min) | 18.8(16.4-21.9) | 19.0(16.5-22.0) | 18.3(16.4-21.9) | .21 |
| MBP (mmHg) | 73.9(68.6-81.8) | 74.7(69.3-82.4) | 72.2(66.5-78.1) | <.001 |
| Heart rate (bpm) | 89.2(79.2-103) | 90.6(79.9-103) | 86.4(76.5-99.0) | .004 |
| SpO_2_ (%) | 97.0(95.5-98.4) | 96.9(95.4-98.4) | 97.3(95.7-98.4) | .28 |
| **Laboratory** |  |  |  |  |
| WBC (K/µL) | 12.8(9.15-18.0) | 13.0(9.20-18.6) | 12.0(8.98-15.7) | .09 |
| RDW (%) | 15.0(13.9-16.6) | 14.9(13.7-16.4) | 15.4(14.4-17.1) | <.001 |
| Platelet (K/µL) | 176(113-235) | 176(112-235) | 175(119-234) | .93 |
| Hemoglobin (g/dL) | 9.60(8.40-11.0) | 9.70(8.40-11.2) | 9.20(8.10-10.4) | .001 |
| Anion gap (mmol/L) | 14.0(12.0-17.0) | 14.0(12.0-16.0) | 15.0(12.0-17.0) | .005 |
| BUN (mg/dL) | 21.0(14.0-34.0) | 19.0(13.0-30.0) | 30.0(18.2-40.8) | <.001 |
| Creatinine (mg/dL) | 1.10(0.80-1.60) | 1.00(0.80-1.50) | 1.45(1.00-1.81) | <.001 |
| Calcium (mg/dL) | 8.10(7.70-8.60) | 8.10(7.70-8.60) | 8.12(7.70-8.60) | .42 |
| Chloride (mEq/L) | 105(101-109) | 105(101-109) | 105(101-108) | .92 |
| Sodium (mEq/L) | 138(135-141) | 138(135-141) | 139(135-141) | .42 |
| Potassium (mEq/L) | 4.20(3.80-4.50) | 4.10(3.80-4.50) | 4.20(3.80-4.60) | .68 |
| **Severity scores** |  |  |  |  |
| SAPS II | 37.0(30.0-47.0) | 35.0(28.0-44.0) | 43.0(37.0-54.0) | <.001 |
| OASIS | 33.0(27.0-39.5) | 32.0(27.0-39.0) | 34.0(28.0-41.0) | .07 |
| APS III | 47.0(35.0-63.5) | 46.0(34.0-63.0) | 51.0(38.0-69.6) | .002 |
| SOFA | 2.00(1.00-5.00) | 2.00(1.00-5.00) | 3.00(1.00-5.00) | .001 |
| CCI | 5.00(3.00-7.00) | 4.00(2.00-6.00) | 9.00(8.00-10.0) | <.001 |
| **Medication, n (%)** |  | | | |
| Ondansetron: | 475 (55.0) | 370 (55.0) | 105 (55.3) | 1.000 |
| Neostigmine: | 101 (11.7) | 87 (13) | 14 (7) | .048 |
| **Events** |  | | | |
| Los hospital, days | 14.6(9.72–23.1) | 14.6(9.61-23.9) | 15.0(10.5-20.8) | .59 |
| Los ICU, days | 3.65(1.94-7.85) | 3.76(1.94-7.91) | 3.30(1.96-7.61) | .35 |
| Hospital mortality, n% | 157 (18.2) | 106 (15.8) | 51 (27) | .001 |
| ICU mortality, n% | 76 (9) | 56 (8) | 20 (11) | .42 |
| 28-day mortality, n% | 171 (19.8) | 107 (15.9) | 64 (34) | <.001 |
| 90-day mortality, n% | 233 (27.0) | 147 (21.8) | 86 (45) | <.001 |
| ^a^Continuous data are presented as median (interquartile range), whereas categorical data are presented as frequency (percentage).  Abbreviations: CHF, congestive heart failure; RR, Respiratory Rate; MBP, Mean Blood Pressure; SpO_2_, Saturation of Peripheral Oxygen; WBC, white blood cell; RDW, red cell distribution width; BUN, blood urea nitrogen; SAPS II, simplified acute physiological score II; OASIS, Oxford Acute Severity of Illness Score; APS III, Acute Physiology Score III; SOFA, sequential organ failure assessment; CCI, Charlson Comorbidity Index; ICU, intensive care unit. | | | | |

**Supplementary Table S3.** Cox proportional hazard ratios (HRs) for all-cause ICU and 90-day mortality in patients with paralytic ileus

| Categories | Model 1 | | | Model 2 | | | Model 3 | | |
| --- | --- | --- | --- | --- | --- | --- | --- | --- | --- |
|  | HR  (95% CI) | *P*-  value | *P* for trend | HR  (95% CI) | *P*-  value | *P* for trend | HR  (95% CI) | *P*-  value | *P* for trend |
| **ICU mortality** |  | | | | | | | | |
| Continuous Variable | 1.161(1.069-1.261) | <.001 |  | 1.142(1.035-1.260) | .008 |  | 1.216(1.060-1.395) | .005 |  |
| Per unit Quartile ^a^ |  |  | .02 |  |  | .23 |  |  | .23 |
| Q1(n=190) | Ref |  |  |  |  |  | Ref |  |  |
| Q2(n=162) | 1.448(0.768-2.729) | .25 |  | 1.204(0.603-2.402) | .60 |  | 1.222(0.577-2.589) | .60 |  |
| Q3(n=159) | 1.765(0.899-3.467) | .10 |  | 1.370(0.624-3.008) | .43 |  | 1.515(0.583-3.941) | .39 |  |
| Q4(n=170) | 2.087(1.088-4.001) | .03 |  | 1.577(0.735-3.384) | .24 |  | 1.892(0.653-5.480) | .24 |  |
| **90-day mortality** |  | | | | | | | | |
| Continuous Variable | 1.203(1.153-1.256) | <.001 |  | 1.230(1.170-1.293) | <.001 |  | 1.249(1.161-1.343) | <.001 |  |
| Per unit Quartile ^a^ |  |  | <.001 |  |  | <0.001 |  |  | .02 |
| Q1(n=190) | Ref |  |  |  |  |  | Ref |  |  |
| Q2(n=162) | 3.370(1.545-3.636) | <.001 |  | 2.679(1.696-4.232) | <.001 |  | 1.922(1.193-3.097) | .007 |  |
| Q3(n=159) | 3.069(1.982-4.753) | <.001 |  | 3.553(2.195-5.750) | <.001 |  | 2.366(1.378-4.065) | .002 |  |
| Q4(n=170) | 4.704(3.134-7.062) | <.001 |  | 5.625(3.512-9.010) | <.001 |  | 3.994(2.224-7.173) | <.001 |  |
| Model 1: unadjusted.  Model 2: adjusted for sex, age.  Model 3: adjusted for sex, age, CHF, renal disease, malignant cancer, sepsis, diabetes, hypertension, RR, SpO_2_, WBC, RDW, platelet, anion gap, BUN, creatinine, SAPS II, OASIS, APS III, SOFA.  a CCI: Q1 (0–3), Q2 (3–5), Q3 (5–7), Q4 (7–14)  Abbreviations: CI, confidence interval; ICU, intensive care unit; CHF, congestive heart failure; RR, Respiratory Rate; SpO_2_, Saturation of Peripheral Oxygen; WBC, white blood cell; RDW, red cell distribution width; BUN, blood urea nitrogen; SAPS II, simplified acute physiological score II; OASIS, Oxford Acute Severity of Illness Score; APS III, Acute Physiology Score III; SOFA, sequential organ failure assessment; CCI, Charlson Comorbidity Index; | | | | | | | | | |

**Supplementary Table S4**. Association of Charlson Comorbidity Index and all-cause mortality outcomes after excluding patients who died within three days of ICU admission

| Outcomes | Group | HR (95%CI) | *P*-value | *P* for trend |
| --- | --- | --- | --- | --- |
| Hospital mortality |  |  |  | .009 |
|  | Q1 | Reference |  |  |
|  | Q2 | 1.66(0.94,2.94) | .08 |  |
|  | Q3 | 1.70(0.85,3.40) | .13 |  |
|  | Q4 | 2.86(1.36,6.04) | .006 |  |
| ICU mortality |  |  |  | .43 |
|  | Q1 | Reference |  |  |
|  | Q2 | 1.33(0.59,2.97) | .49 |  |
|  | Q3 | 1.48(0.51,4.32) | .47 |  |
|  | Q4 | 1.66(0.50,5.44) | .41 |  |
| 28-day mortality |  |  |  | <.001 |
|  | Q1 | Reference |  |  |
|  | Q2 | 2.07(1.15,3.72) | .02 |  |
|  | Q3 | 2.52(1.29,4.93) | .007 |  |
|  | Q4 | 4.37(2.13,8.99) | <.001 |  |
| 90-day mortality |  |  |  | <.001 |
|  | Q1 | Reference |  |  |
|  | Q2 | 2.09(1.28,3.42) | .003 |  |
|  | Q3 | 2.48(1.41,4.34) | .001 |  |
|  | Q4 | 4.35(2.37,7.97) | <.001 |  |
| Note: Q1 (0–3), Q2 (3–5), Q3 (5–7), Q4 (7–14).  Cox proportional hazards model was adjusted for sex, age, CHF, renal disease, malignant cancer, sepsis, diabetes, hypertension, RR, SpO_2_, WBC, RDW, platelet, anion gap, BUN, creatinine, SAPS II, OASIS, APS III, SOFA.  Abbreviations: CI, confidence interval; ICU, intensive care unit; CHF, congestive heart failure; RR, Respiratory Rate; SpO_2_, Saturation of Peripheral Oxygen; WBC, white blood cell; RDW, red cell distribution width; BUN, blood urea nitrogen; SAPS II, simplified acute physiological score II; OASIS, Oxford Acute Severity of Illness Score; APS III, Acute Physiology Score III; SOFA, sequential organ failure assessment. | | | | |

**Supplementary Table S5**. Association of Charlson Comorbidity Index and all-cause mortality outcomes after stratification by CCI threshold (≤ 4.5 vs. > 4.5)

| Outcomes | Group | HR (95%CI) | *P* |
| --- | --- | --- | --- |
| Hospital mortality |  |  |  |
|  | CCI ≤ 4.5 | Reference |  |
|  | CCI > 4.5 | 2.06(1.29,3.29) | .002 |
| ICU mortality |  |  |  |
|  | CCI ≤ 4.5 | Reference |  |
|  | CCI > 4.5 | 1.94(0.97,3.90) | .06 |
| 28-day mortality |  |  |  |
|  | CCI ≤ 4.5 | Reference |  |
|  | CCI > 4.5 | 2.31(1.46,3.69) | <.001 |
| 90-day mortality |  |  |  |
|  | CCI ≤ 4.5 | Reference |  |
|  | CCI > 4.5 | 2.01(1.36,2.97) | <.001 |
| Note: Q1 (0–3), Q2 (3–5), Q3 (5–7), Q4 (7–14).  Cox proportional hazards model was adjusted for sex, age, CHF, renal disease, malignant cancer, sepsis, diabetes, hypertension, RR, SpO_2_, WBC, RDW, platelet, anion gap, BUN, creatinine, SAPS II, OASIS, APS III, SOFA.  Abbreviations: CI, confidence interval; ICU, intensive care unit; CHF, congestive heart failure; RR, Respiratory Rate; SpO_2_, Saturation of Peripheral Oxygen; WBC, white blood cell; RDW, red cell distribution width; BUN, blood urea nitrogen; SAPS II, simplified acute physiological score II; OASIS, Oxford Acute Severity of Illness Score; APS III, Acute Physiology Score III; SOFA, sequential organ failure assessment. | | | |

**Supplementary Table S6**. Sensitivity analysis for the relationships between Charlson Comorbidity Index and all-cause mortality outcomes stratified by SOFA (≤1 vs. 2-4 vs. >4)

| Outcomes | Group | HR (95%CI) | *P* | *P* for trend |
| --- | --- | --- | --- | --- |
| **Hospital mortality** |  |  |  | .12 |
| SOFA ≤1 |  |  |  |  |
|  | Q1 | Reference |  |  |
|  | Q2 | 4.00(0.79, 20.19) | .09 |  |
|  | Q3 | 5.05(0.89, 28.76) | .07 |  |
|  | Q4 | 21.88(3.12, 153.26) |  |  |
| 1 < SOFA ≤ 4 |  |  |  |  |
|  | Q1 | Reference |  |  |
|  | Q2 | 1.27 (0.31, 5.14) | .74 |  |
|  | Q3 | 4.72 (1.20, 18.56) | .03 |  |
|  | Q4 | 5.69 (1.09, 29.73) | .04 |  |
| SOFA > 4 |  |  |  |  |
|  | Q1 | Reference |  |  |
|  | Q2 | 1.49(0.54, 4.11) | .44 |  |
|  | Q3 | 2.10(0.72, 6.12) | .17 |  |
|  | Q4 | 2.79 (0.89, 8.75) | .08 |  |
| **ICU mortality** |  |  |  | .50 |
| SOFA ≤1 |  |  |  |  |
|  | Q1 | Reference |  |  |
|  | Q2 | 5.62 (0.19, 164.03) | .32 |  |
|  | Q3 | 9.76 (0.18, 527.61) | .26 |  |
|  | Q4 | 235.77(4.13, 13454.52) | .01 |  |
| 1 < SOFA ≤ 4 |  |  |  |  |
|  | Q1 | Reference |  |  |
|  | Q2 | 4.23 (0.40, 44.26) | .23 |  |
|  | Q3 | 15.59 (1.02, 237.77) | .05 |  |
|  | Q4 | 9.34 (0.93, 93.33) | .06 |  |
| SOFA > 4 |  |  |  |  |
|  | Q1 | Reference |  |  |
|  | Q2 | 1.38 (0.38, 5.06) | .63 |  |
|  | Q3 | 2.08 (0.48, 9.02) | .33 |  |
|  | Q4 | 1.49 (0.29, 7.60) | .63 |  |
| **28-day mortality** |  |  |  | .23 |
| SOFA ≤1 |  |  |  |  |
|  | Q1 | Reference |  |  |
|  | Q2 | 5.65 (1.20, 26.67) | .03 |  |
|  | Q3 | 8.29 (1.61, 42.64) | .01 |  |
|  | Q4 | 21.29 (3.54, 128.03) | .007 |  |
| 1 < SOFA ≤ 4 |  |  |  |  |
|  | Q1 | Reference |  |  |
|  | Q2 | 1.56 (0.41, 6.00) | .51 |  |
|  | Q3 | 3.51 (0.90, 13.79) | .07 |  |
|  | Q4 | 5.48 (1.08, 27.94) | .04 |  |
| SOFA > 4 |  |  |  |  |
|  | Q1 | Reference |  |  |
|  | Q2 | 1.39 (0.49, 3.90) | .54 |  |
|  | Q3 | 2.19 (0.72, 6.68) | .17 |  |
|  | Q4 | 2.90 (0.89, 9.45) | .08 |  |
| **90-day mortality** |  |  |  | .08 |
| SOFA ≤1 |  |  |  |  |
|  | Q1 | Reference |  |  |
|  | Q2 | 5.53 (1.45, 21.05) | .01 |  |
|  | Q3 | 6.00 (1.45, 24.84) | .01 |  |
|  | Q4 | 21.25 (4.49, 100.66) | .008 |  |
| 1 < SOFA ≤ 4 |  |  |  |  |
|  | Q1 | Reference |  |  |
|  | Q2 | 2.64 (0.89, 7.86) | .08 |  |
|  | Q3 | 5.28 (1.72, 16.22) | .04 |  |
|  | Q4 | 10.65 (2.81, 40.33) | .007 |  |
| SOFA > 4 |  |  |  |  |
|  | Q1 | Reference |  |  |
|  | Q2 | 1.36 (0.59, 3.12) | .47 |  |
|  | Q3 | 2.21 (0.89, 5.50) | .09 |  |
|  | Q4 | 2.97 (1.09, 8.10) | .03 |  |
| Note: Q1 (0–3), Q2 (3–5), Q3 (5–7), Q4 (7–14).  Cox proportional hazards model was adjusted for sex, age, CHF, renal disease, malignant cancer, sepsis, diabetes, hypertension, RR, SpO_2_, WBC, RDW, platelet, anion gap, BUN, creatinine, SAPS II, OASIS, APS III, SOFA.  Abbreviations: CI, confidence interval; ICU, intensive care unit; CHF, congestive heart failure; RR, Respiratory Rate; SpO_2_, Saturation of Peripheral Oxygen; WBC, white blood cell; RDW, red cell distribution width; BUN, blood urea nitrogen; SAPS II, simplified acute physiological score II; OASIS, Oxford Acute Severity of Illness Score; APS III, Acute Physiology Score III; SOFA, sequential organ failure assessment. | | | | |

| **Supplementary Table S7.** Baseline characteristics of ICU patients with paralytic ileus in the training and test cohorts for machine learning model development ^a^ | | | | |
| --- | --- | --- | --- | --- |
| Variables | Total (n = 863) | Test Set  (n = 261) | Training Set  (n = 602) | *P*-value |
| **Sex, n (%)** |  |  |  | .71 |
| Female | 288 (33) | 90 (34) | 198 (33) |  |
| Male | 575 (67) | 171 (66) | 404 (67) |  |
| Age (years) | 65.42 (54.56-75.5) | 66.02 (56.19-75.57) | 65.34 (54.1-75.42) | .67 |
| **Race, n (%)** |  |  |  | .79 |
| White | 576 (67) | 172 (66) | 404 (67) |  |
| Others | 287 (33) | 89 (34) | 198 (33) |  |
| **Comorbidities, n (%)** |  | | | |
| CHF, n (%) |  |  |  | .73 |
| No | 643 (75) | 197 (75) | 446 (74) |  |
| Yes | 220 (25) | 64 (25) | 156 (26) |  |
| Renal disease, n (%) |  |  |  | .7 |
| No | 669 (78) | 205 (79) | 464 (77) |  |
| Yes | 194 (22) | 56 (21) | 138 (23) |  |
| Malignant cancer,  n (%) |  |  |  | .54 |
| No | 702 (81) | 216 (83) | 486 (81) |  |
| Yes | 161 (19) | 45 (17) | 116 (19) |  |
| Sepsis, n (%) |  |  |  | .22 |
| No | 581 (67) | 184 (70) | 397 (66) |  |
| Yes | 282 (33) | 77 (30) | 205 (34) |  |
| Diabetes, n (%) |  |  |  | .98 |
| No | 637 (74) | 192 (74) | 445 (74) |  |
| Yes | 226 (26) | 69 (26) | 157 (26) |  |
| Hypertension, n (%) |  |  |  | .14 |
| No | 508 (59) | 164 (63) | 344 (57) |  |
| Yes | 355 (41) | 97 (37) | 258 (43) |  |
| **Vital signs** |  | | | |
| RR (breaths/min) | 18.76 (16.44-21.95) | 18.82 (16.64-21.78) | 18.71 (16.32-22.13) | .93 |
| MBP (mmHg) | 73.95 (68.61-81.8) | 74.06 (68.8-82) | 73.95 (68.56-81.46) | .88 |
| Heart rate (bpm) | 89.24 (79.15-102.73) | 89.14 (79-101.83) | 89.25 (79.19-102.78) | .98 |
| SpO_2_ (%) | 97 (95.46-98.38) | 96.9 (95.18-98.29) | 97 (95.59-98.43) | .25 |
| WBC (K/µL) | 12.8 (9.15-18) | 13 (9.2-18.4) | 12.7 (9.1-17.78) | .60 |
| RDW (%) | 15 (13.85-16.6) | 14.8 (13.7-16.4) | 15.2 (14-16.6) | .06 |
| Platelet (K/µL) | 176 (113-235) | 179 (115-247.43) | 173 (113-232.25) | .31 |
| Hemoglobin (g/dL) | 9.6 (8.4-11) | 9.6 (8.4-11.2) | 9.6 (8.4-11) | .39 |
| Anion gap (mmol/L) | 14 (12-17) | 14 (12-16) | 14 (12-17) | .31 |
| BUN (mg/dL) | 21 (14-34) | 21 (14-31) | 21.5 (14-35.45) | .47 |
| Creatinine (mg/dL) | 1.1 (0.8-1.6) | 1 (0.8-1.5) | 1.1 (0.8-1.64) | .02 |
| Calcium (mg/dL) | 8.1 (7.7-8.6) | 8.1 (7.7-8.6) | 8.1 (7.7-8.6) | .95 |
| Chloride (mEq/L) | 105 (101-109) | 105 (101-108) | 104.11(101-109) | .69 |
| Sodium (mEq/L) | 138 (135-141) | 138 (135-141) | 138 (135-141) | .59 |
| Potassium (mEq/L) | 4.2 (3.8-4.5) | 4.1 (3.7-4.5) | 4.2 (3.8-4.6) | .19 |
| **Severity scores** |  | | | |
| SAPS II | 37 (30-47) | 36 (28-47) | 37 (30-47) | .43 |
| OASIS | 33 (27-39.5) | 32 (26-39) | 33 (28-40) | .23 |
| APS III | 47 (35-63.5) | 46 (34-63) | 47 (35-63.75) | .53 |
| SOFA | 2 (1-5) | 2 (1-4.72) | 2.45 (1-5) | .13 |
| CCI | 5 (3-7) | 5 (3-7) | 5 (3-7) | .54 |
| **Medication, n (%)** |  | | | |
| Ondansetron, n (%) |  |  |  | .04 |
| No | 388 (45) | 103 (39) | 285 (47) |  |
| Yes | 475 (55) | 158 (61) | 317 (53) |  |
| Neostigmine, n (%) |  |  |  | .65 |
| No | 762 (88) | 228 (87) | 534 (89) |  |
| Yes | 101 (12) | 33 (13) | 68 (11) |  |
| **Event** |  | | | |
| Hospital mortality,  n (%) |  |  |  | 1.00 |
| No | 706 (82) | 214 (82) | 492 (82) |  |
| Yes | 157 (18) | 47 (18) | 110 (18) |  |
| ^a^ Continuous data are presented as median (interquartile range), whereas categorical data are presented as frequency (percentage)  Abbreviations: CHF, congestive heart failure; RR, Respiratory Rate; MBP, Mean Blood Pressure; SpO_2_, Saturation of Peripheral Oxygen; WBC, white blood cell; RDW, red cell distribution width; BUN, blood urea nitrogen; SAPS II, simplified acute physiological score II; OASIS, Oxford Acute Severity of Illness Score; APS III, Acute Physiology Score III; SOFA, sequential organ failure assessment; CCI, Charlson Comorbidity Index | | | | |


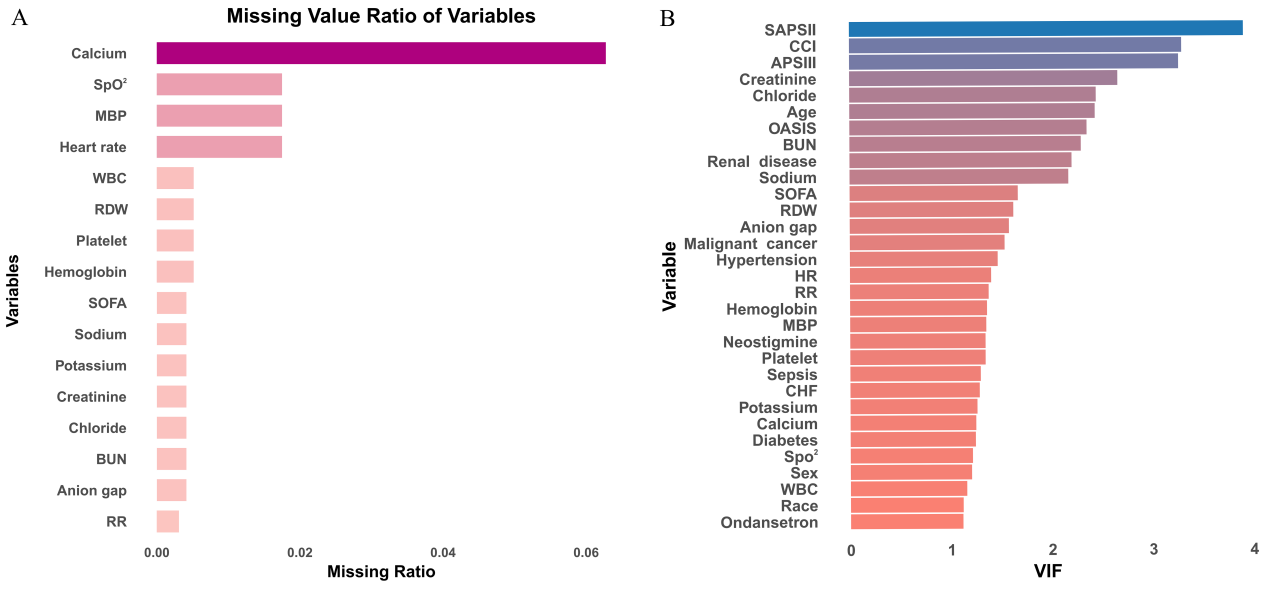


**Supplementary Fig. S1 A** Missing value ratio plot of variables. Note: Variables not shown have a missing rate of 0%. **B** The variance inflation factor (VIF) values for each independent variable. Abbreviations: SAPS II, simplified acute physiological score II; CCI, Charlson Comorbidity Index; APS III, Acute Physiology Score III; OASIS, Oxford Acute Severity of Illness Score; BUN, blood urea nitrogen; SOFA, sequential organ failure assessment; RDW, red cell distribution width; HR, Heart rate; RR, Respiratory Rate; MBP, Mean Blood Pressure; CHF, congestive heart failure; SpO_2_, Saturation of Peripheral Oxygen; WBC, white blood cell.


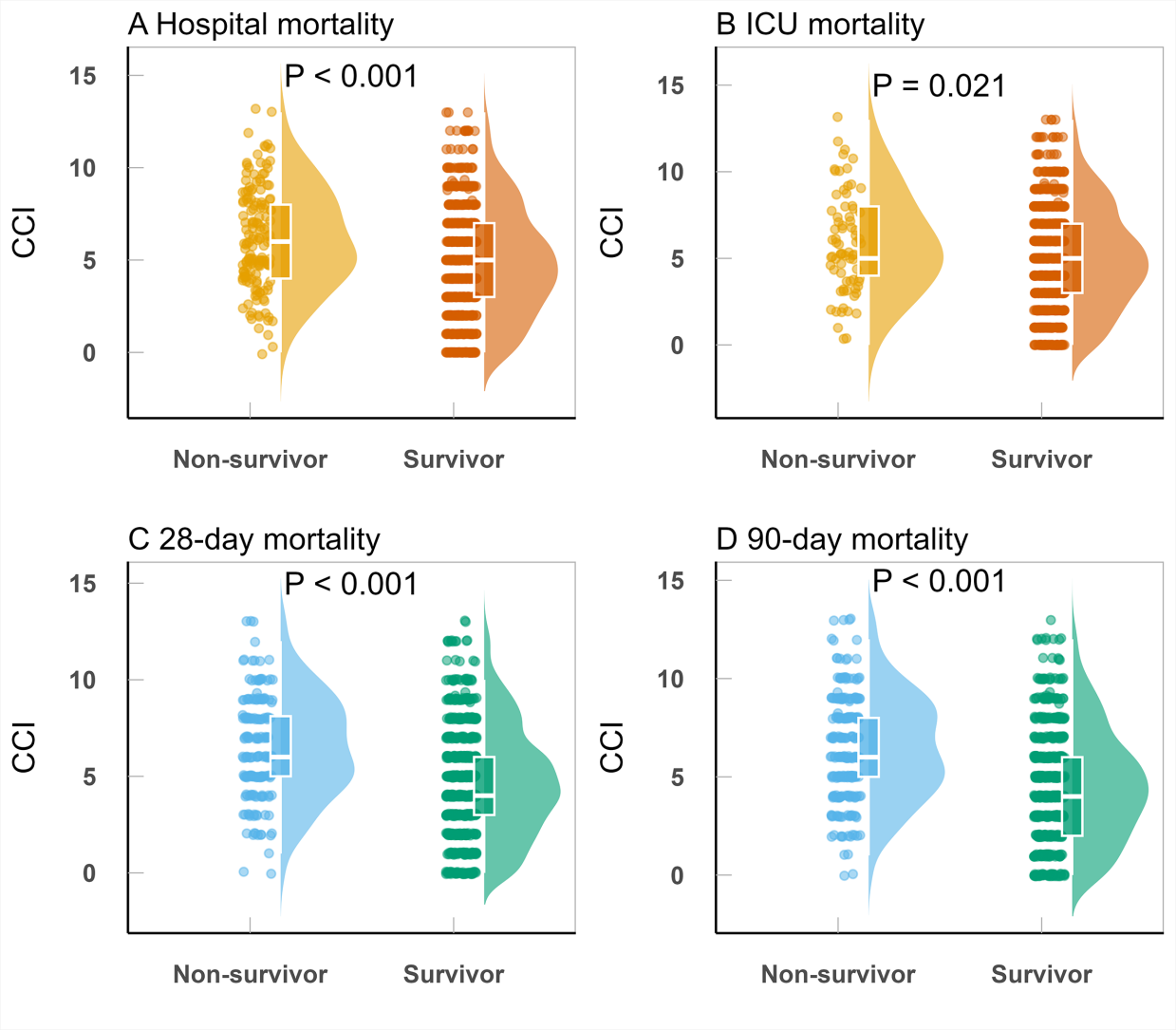


**Supplementary Fig. S2** Distribution of the Charlson Comorbidity Index stratified by mortality status for Hospital (A), ICU (B), 28-day (C), and 90-day (D)


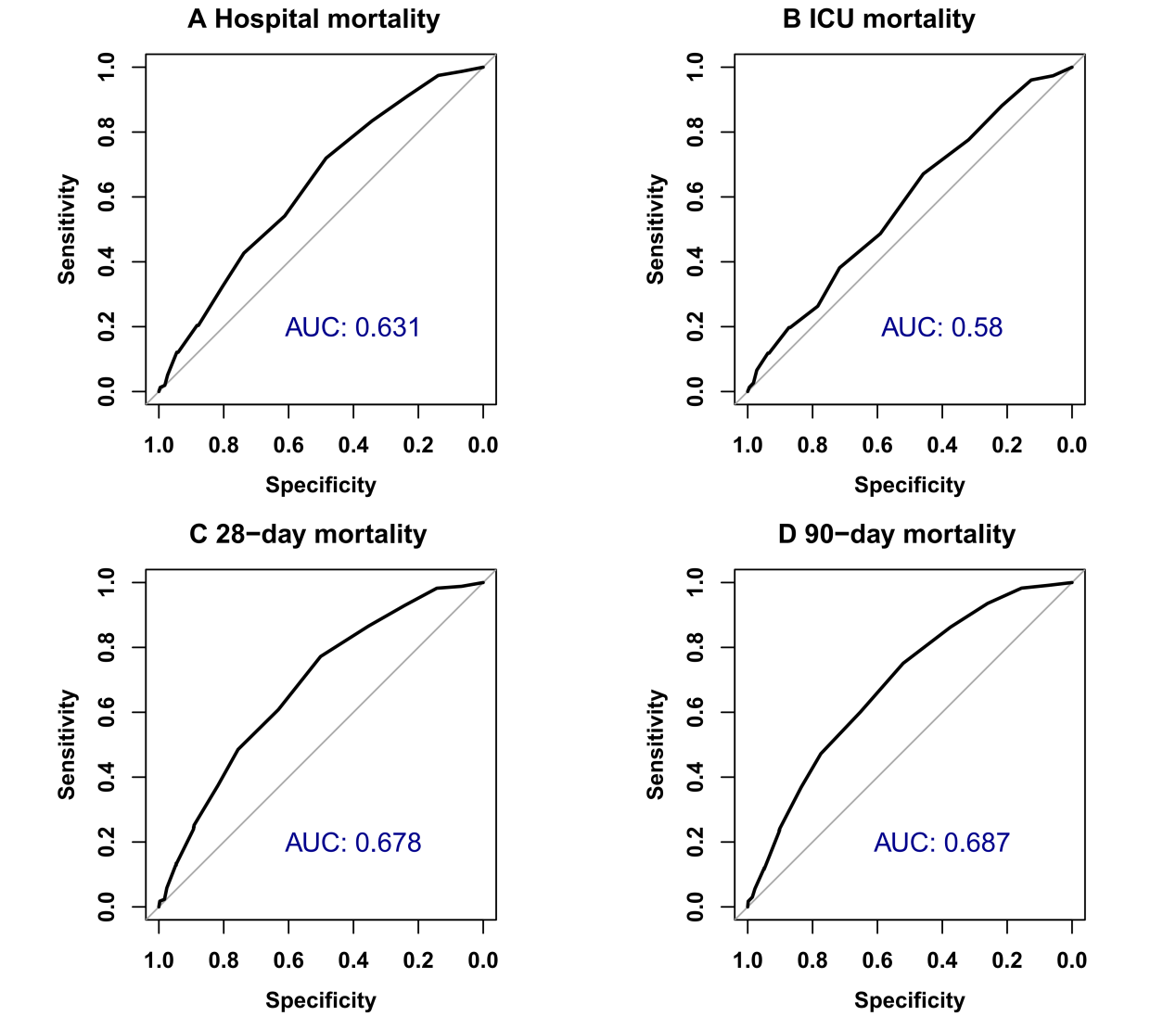


**Supplementary Fig. S3** ROC analysis of Charlson Comorbidity Index for Predicting Mortality: Hospital (A), ICU (B), 28-day (C), 90-day (D)


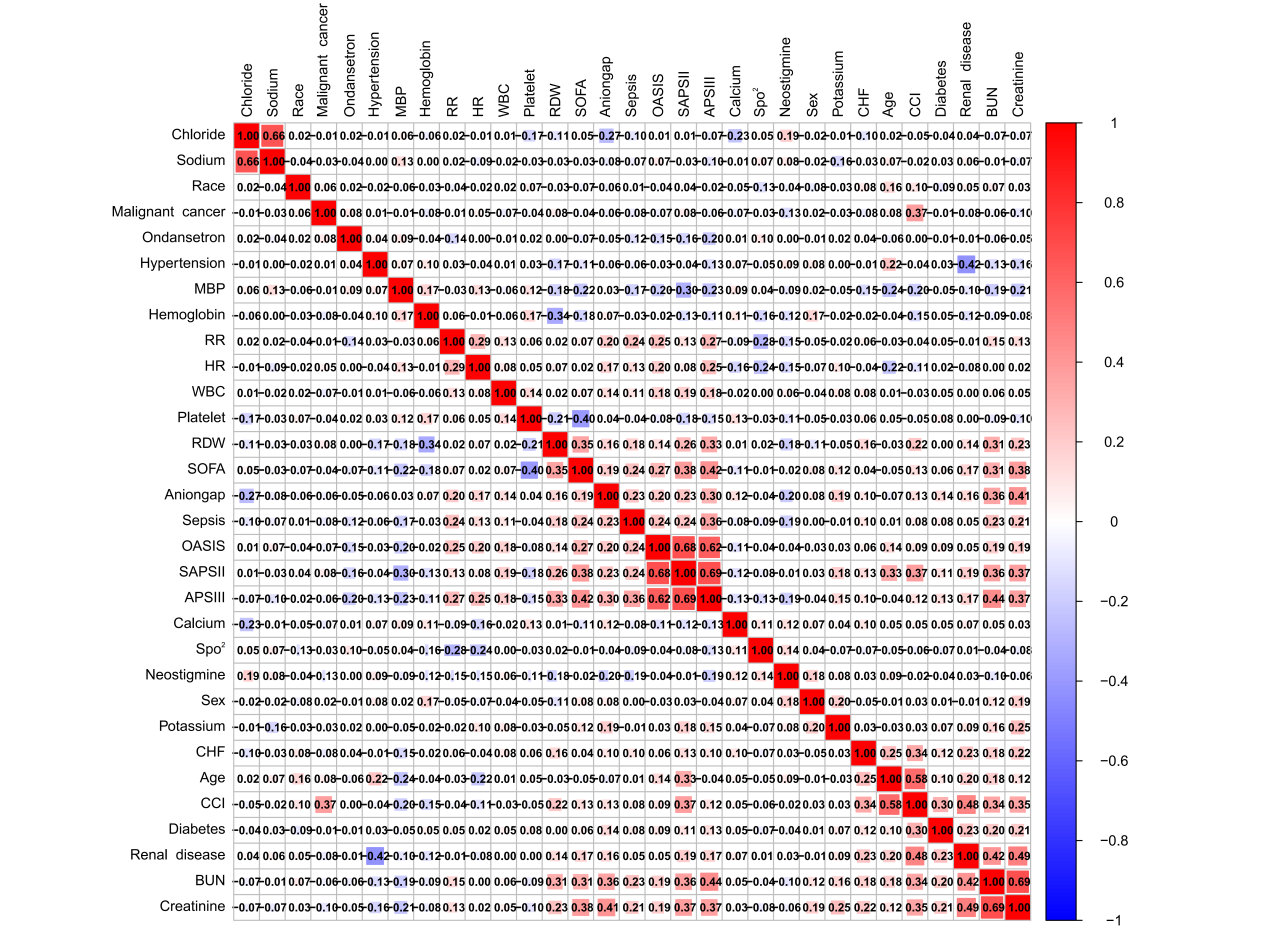


**Supplementary Fig. S4** Correlation heatmap of all included variables.Abbreviations: SAPS II, simplified acute physiological score II; CCI, Charlson Comorbidity Index; APS III, Acute Physiology Score III; OASIS, Oxford Acute Severity of Illness Score; BUN, blood urea nitrogen; SOFA, sequential organ failure assessment; RDW, red cell distribution width; HR, Heart rate; RR, Respiratory Rate; MBP, Mean Blood Pressure; CHF, congestive heart failure; SpO_2_, Saturation of Peripheral Oxygen; WBC, white blood cell.


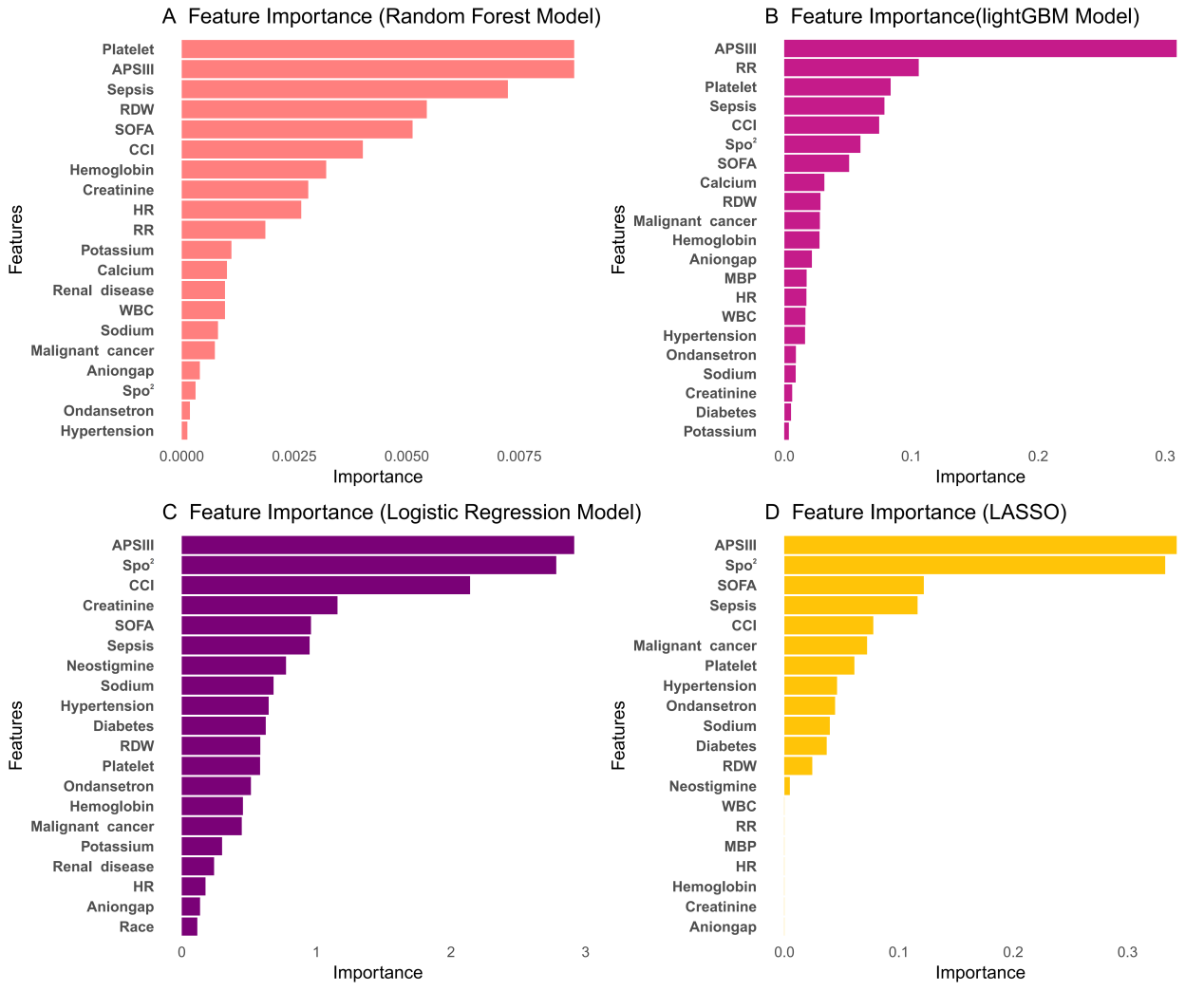


**Supplementary Fig. S5** Feature selection for predicting hospital all-cause mortality after ICU admission in patients with paralytic ileus**.** The feature importance scores were derived using different methods: (A) Random Forest Model, (B) LightGBM Model, (C) Logistic Regression Model, and (D) LASSO Model. Abbreviations: CCI, Charlson Comorbidity Index; APS III, Acute Physiology Score III; SOFA, sequential organ failure assessment; RDW, red cell distribution width; HR, Heart rate; RR, Respiratory Rate; MBP, Mean Blood Pressure; SpO_2_, Saturation of Peripheral Oxygen; WBC, white blood cell.


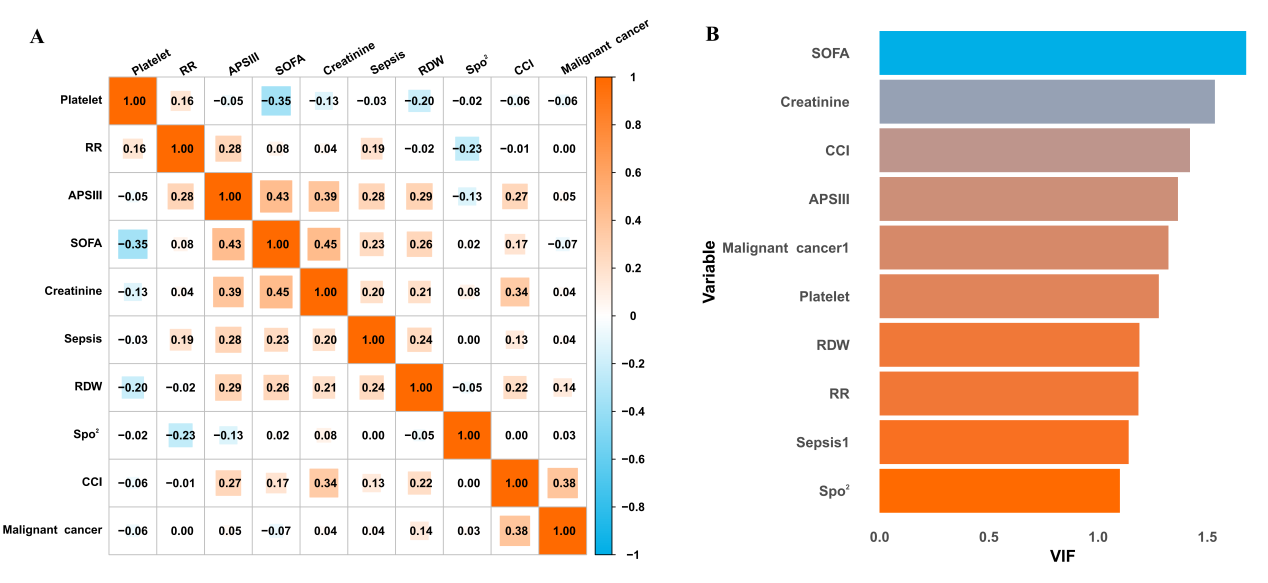


**Supplementary Fig. S6** Pearson’s correlation test (A) and variance inflation factor test (B) for selected features**.** Abbreviations: SOFA, sequential organ failure assessment; CCI, Charlson Comorbidity Index; APS III, Acute Physiology Score III; RDW, red cell distribution width; RR, Respiratory Rate; SpO_2_, Saturation of Peripheral Oxygen.

**
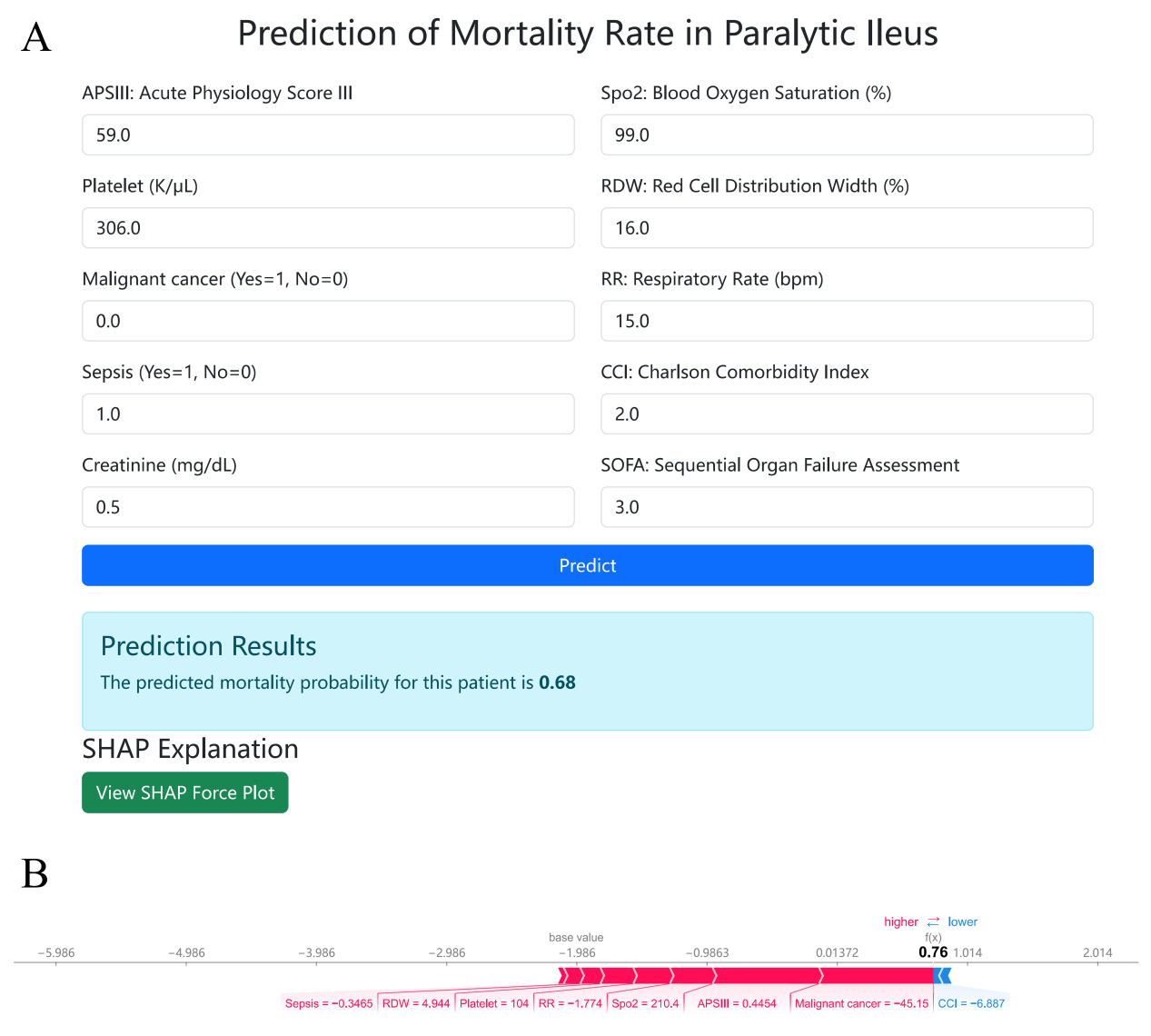
Supplementary Fig. S7** Online platform for the light gradient boosting machine model (A) and SHAP Force Plot (B). This prototype is for research demonstration only and does not replace clinical judgment; no protected health information is stored.
